# Eutherian-Specific Gene *TRIML2* Attenuates Inflammation in the Evolution of Placentation

**DOI:** 10.1093/molbev/msz238

**Published:** 2019-10-09

**Authors:** Xuzhe Zhang, Mihaela Pavlicev, Helen N Jones, Louis J Muglia

**Affiliations:** 1 Division of Human Genetics, Center for Prevention of Preterm Birth, Perinatal Institute, Cincinnati Children’s Hospital Medical Center, Cincinnati, OH; 2 Department of Pediatrics, University of Cincinnati College of Medicine, Cincinnati, OH; 3 March of Dimes Prematurity Research Center Ohio Collaborative, Cincinnati, OH; 4 Division of Pediatric Surgery, Cincinnati Children’s Hospital Medical Center, Cincinnati, OH; 5 Department of Surgery, University of Cincinnati College of Medicine, Cincinnati, OH

**Keywords:** TRIM family protein, Eutheria, placentation, inflammation, endogenous retrovirus, coevolution

## Abstract

Evolution of highly invasive placentation in the stem lineage of eutherians and subsequent extension of pregnancy set eutherians apart from other mammals, that is, marsupials with short-lived placentas, and oviparous monotremes. Recent studies suggest that eutherian implantation evolved from marsupial attachment reaction, an inflammatory process induced by the direct contact of fetal placenta with maternal endometrium after the breakdown of the shell coat, and shortly before the onset of parturition. Unique to eutherians, a dramatic downregulation of inflammation after implantation prevents the onset of premature parturition, and is critical for the maintenance of gestation. This downregulation likely involved evolutionary changes on maternal as well as fetal/placental side. *Tripartite-motif family-like2* (*TRIML2*) only exists in eutherian genomes and shows preferential expression in preimplantation embryos, and trophoblast-derived structures, such as chorion and placental disc. Comparative genomic evidence supports that *TRIML2* originated from a gene duplication event in the stem lineage of Eutheria that also gave rise to eutherian *TRIML1*. Compared with TRIML1, TRIML2 lost the catalytic RING domain of E3 ligase. However, only *TRIML2* is induced in human choriocarcinoma cell line JEG3 with poly(I:C) treatment to simulate inflammation during viral infection. Its knockdown increases the production of proinflammatory cytokines and reduces trophoblast survival during poly(I:C) stimulation, while its overexpression reduces proinflammatory cytokine production, supporting *TRIML2*’s role as a regulatory inhibitor of the inflammatory pathways in trophoblasts. TRIML2’s potential virus-interacting PRY/SPRY domain shows significant signature of selection, suggesting its contribution to the evolution of eutherian-specific inflammation regulation during placentation.

## Introduction

Although both eutherians and marsupials are viviparous and form fully functional placentas during pregnancy, eutherian ancestors evolved highly invasive form of placentation, with subsequent extension of pregnancy. These reproductive characteristics enabled the offspring to be born at more mature stages and set eutherians apart from other mammals (Wildman et al. 2006; Renfree 2010; Chavan et al. 2016). Underlying such remarkable evolutionary achievement unique to eutherian reproduction is the mechanism that enables the semi-allogeneic fetus direct contact with, and breaching of the integrity of maternal tissue without the rejection by the maternal immune system (Chavan et al. 2017). Recent comparative studies in mammals with different reproduction modes have shed light on this conundrum (Griffith et al. 2017).

Though viviparous, marsupials retain many characteristics of the oviparous ancestors that are seen in extant monotremes. In both marsupials and monotremes, the contact between the conceptus and maternal tissue is limited by a layer consisting of the zona pellucida, mucoid coat, and shell coat (Frankenberg and Renfree 2018). In monotremes, the shell coat thickens dramatically as pregnancy proceeds (Frankenberg 2018). In marsupials, instead of thickening, the shell coat breaks down and enables transient direct attachment of the placenta to the endometrium, which triggers maternal inflammation reaction and results in parturition within days. Interestingly, implantation processes in eutherians show molecular signatures consistent with the marsupial attachment reaction, but unlike marsupials, the upregulation of proinflammatory genes does not trigger parturition. Rather, a transition into an anti-inflammatory period occurs, and the low inflammatory status is maintained until the end of the extended pregnancy (Griffith et al. 2017).

The preservation and modification of the inflammatory reaction associated with implantation throughout evolution suggest that it is a critical process. Indeed, evidence supports that inflammation is constitutive for the establishment of pregnancy, due to its pregnancy-enhancing aspects in processes such as angiogenesis (Chaouat 2013). On the other hand, excessive inflammation, on maternal and/or fetal/placental sides, is associated with adverse reproductive outcomes, such as fetal loss (Zenclussen et al. 2003; Calleja-Agius et al. 2012). Thus, the evolution in eutherians of mechanisms to tightly control the nature and extent of inflammation both temporally and spatially was crucial to enable the transition to the anti-inflammatory status unique to eutherian prolonged pregnancies.

Maternal-fetal tolerance is not the only source of selective pressure on the eutherian immune regulation during pregnancy. Other important sources are infectious pathogens. Intrauterine infection lowers maternal reproductive fitness (Mor 2008). Expansion of gene families involved in immune response often accompanies such arms races between hosts and pathogens (Worobey et al. 2007). One gene family that shows such rapid expansion is the tripartite motif (TRIM) family, thus named due to the N-terminal RBCC (RING, B-box, and coiled-coil) motif (Cao et al. 1997). Although each of these three submotifs also exists independently in plants, the order of combination is conserved since early in metazoan evolution. Afterward, the RBCC domain has been associated with several different C-terminal domains; however, the RBCC domain per se has been evolving as a single block without domain-swapping between TRIMs. There is no evidence for domain acquisition in RBCC motif, however domain loss that results in the lack of at least one of the three N-terminal domains has happened multiple times, and has given rise to the noncanonical TRIM-like members of this protein family. Based on their protein structure and evolutionary history, TRIMs can be divided into two groups. Group-1 TRIMs possess a variety of C-terminal motifs and are shared by invertebrates and vertebrates, and group-2 TRIMs use SPRY domain as the core C-terminal motif and can only be found in vertebrate genomes. The association of N-terminal RBCC with C-terminal SPRY domain is followed by rapid evolution and gene expansion through gene duplication in vertebrate genomes (Sardiello et al. 2008). The first major expansion of group-2 TRIMs co-occurred with the evolution of the adaptive immunity in jawed vertebrates. Subsequent expansions often paralleled major developments of the immune system, especially the evolution of an ever more complex interferon pathway (Versteeg et al. 2014). Functionally, many TRIMs can be induced by the activation of pattern-recognition receptors during infection, and through their E3 ligase activity, regulate the production of proinflammatory cytokines, thus influence the course of inflammation and innate immunity (Ozato et al. 2008). Through gene duplication, the TRIM family can function as a reservoir to provide new TRIM genes to fine-tune the inflammation/immune response.

As the TRIM family shows rapid expansion in eutherian genomes, we hypothesized that some of these eutherian-specific TRIMs, especially those with preferential expression at the maternal-fetal interface, may contribute to the establishment of the eutherian-specific anti-inflammatory phase. To test this hypothesis, we carried out comparative genomic studies and gene expression profiling, and found that *tripartite-motif family-like 2* (*TRIML2*) only exists in eutherian genomes and is expressed preferentially in preimplantation embryos, chorion, and placental disc. Functional study in human choriocarcinoma cell line JEG3 supports that *TRIML2* reduces proinflammatory cytokine production in trophoblasts. Multiple sites of TRIML2 PRY/SPRY domain, which have been demonstrated to contribute to retrovirus restriction function of other TRIMs, show significant signature of positive selection, supporting the notion that *TRIML2* may have contributed to the evolution of inflammation regulation during eutherian placentation.

## Results

### *TRIML2* Exists in All Extant Eutherian Superorders but Is Absent in Marsupial and Monotreme Genomes

**Fig. 1.**
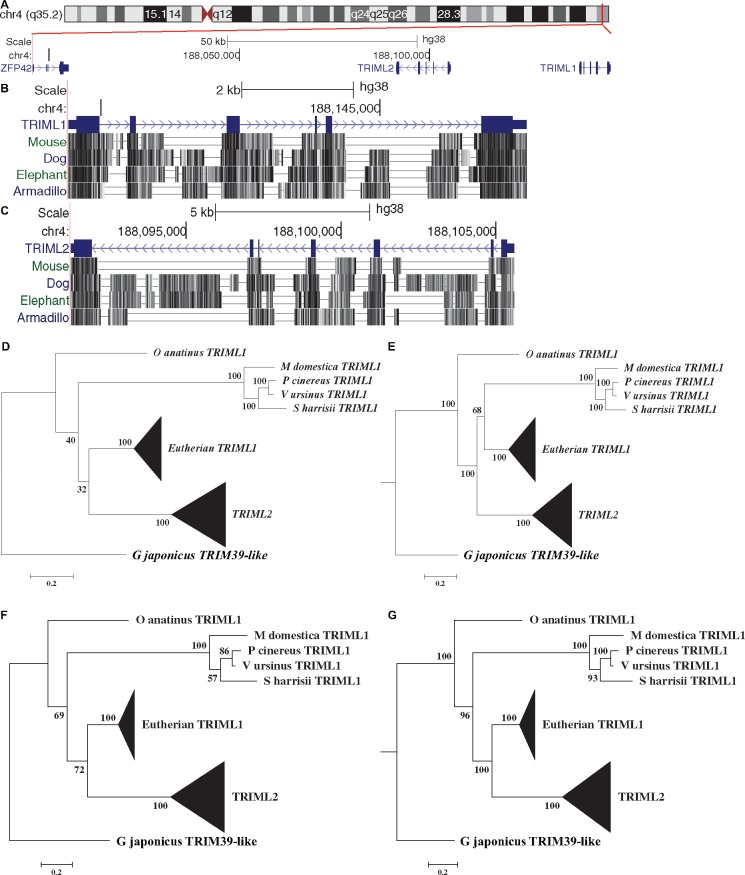
*TRIML2* likely resulted from a gene duplication event in eutherian stem lineage. (*A*) Human *TRIML1* and *TRIML2* are located at the telomeric end of the long arm of chromosome 4. (*B* and *C*) Multiple alignments of *TRIML1* and *TRIML2* loci show both genes exist in all four superorders of eutherian. (*D–G*) Phylogenetic trees generated through maximum likelihood (*D*, mRNA tree; *F*, protein tree) and Bayesian analyses (*E*, mRNA tree; *G*, protein tree), with bootstrap percentages/posterior probabilities.

To study the role TRIMs played during the evolution of eutherian placentation, we looked for TRIMs that likely evolved in the stem lineage of eutherians, that is, after the separation of marsupials but before the branching of the four extant eutherian superorders. As many TRIMs, especially vertebrate-specific group-2 TRIMs, exist in spatial gene clusters (Versteeg et al. 2014), we scanned for TRIM clusters that exist in all four representative species of extant eutherian superorders though UCSC genome browser, and found that *TRIML1* and *TRIML2* at the telomeric end of the long arm of human chromosome 4 exist in all four representative species (fig. 1*A–C*). We downloaded protein sequences of TRIML1 and TRIML2 of all eutherians with well-documented placentation interdigitation and invasiveness (56 species, supplementary fig. S1, Supplementary Material online), and verified through BLAST that TRIML1 proteins of any two eutherian species always find each other as the best hit in the opposite genome with e-value 0.0. Similarly, eutherian TRIML2 proteins also meet the Reciprocal Best Hits (RBH) criterion for ortholog detection (Moreno-Hagelsieb and Latimer 2008). This evidence supports the existence of TRIML1 and TRIML2 orthologs in all four extant eutherian superorders, which suggests that both genes evolved before the separation of the four extant eutherian superorders.

To help estimate the gene age of *TRIML1* and *TRIML2*, we used human TRIML1 and TRIML2 as queries and performed Blastp searches against nonredundant protein sequence databases of taxa Reptilia, Monotremata, and Marsupialia. In reptilian genomes, no genes are annotated as *TRIML1*, and the most similar genes of both human TRIML1 and TRIML2 are TRIM39-like proteins. However, reptilian TRIM39-like proteins show highest similarity with eutherian TRIM39 in the reciprocal search rather than eutherian TRIML1 or TRIML2, supporting the lack of orthologs of either TRIML1 or TRIML2 in Reptilia.

**Fig. 2.**
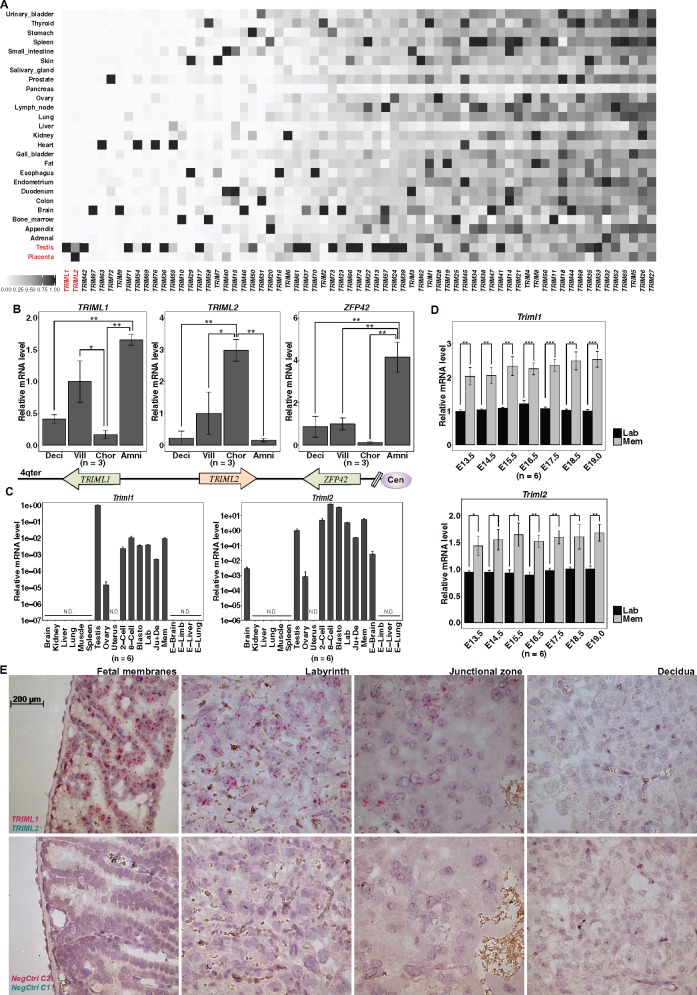
*TRIML1* and *TRIML2* show different restricted expression patterns. (*A*) Heatmap of relative tissue expression levels of human *TRIM* genes, showing restricted expression of *TRIML1* and *TRIML2* (data from the Human Protein Atlas, expression level in each tissue normalized to the highest RPKM for each *TRIM* gene, supplementary fig. S4, Supplementary Material online). (*B*) Preferential expression compartment of *TRIML1*, *TRIML2*, and *ZFP42* in extraembryonic tissues correlates with their transcribing direction. Expression levels normalized to placenta villi. Values are mean ± SEM. One-way ANOVA, Tukey’s HSD, **P < 0.01, *P < 0.05. Deci, placental decidua; Vill, placental villi; Chor, chorion; Amni, amnion; Cen, centromere; 4qter, terminal of chromosome 4 long arm; arrows indicate the transcription direction of each gene. (*C*) *Triml1* and *Triml2* expression in adult and embryo mouse tissue show restricted expression in preimplantation embryo, extraembryonic tissues, testis, and ovary. Expression levels normalized to placental villi. N.D., not detected; 2-Cell, 2-cell stage embryo; 8-Cell, 8-cell stage embryo; Blasto, blastocyst; Lab, E15.5 labyrinth; Ju+De, E15.5 junctional zone, and decidua; Mem, E15.5 fetal membranes; E-Brain, E15.5 embryo brain; E-Limb, E15.5 embryo limb; E-Liver, E15.5 embryo liver; E-Lung, E15.5 embryo lung. (*D*) *Triml1* and *Triml2* show higher expression in fetal membrane than in placental labyrinth. Expression levels normalized to E19.0 labyrinth. Values are mean ± SEM. *t*-Test, ***P < 0.001, **P < 0.01, *P < 0.05. (*E*) RNAScope assay showing *Triml1* and *Triml2* expression in extraembryonic structures, upper row: signal from *Triml1* and *Triml2*-specific probes, lower row: nonspecific signals from negative control probes.

In monotreme and marsupial genomes, the proteins most similar to human TRIML1 and TRIML2 are both protein products of genes annotated as probable *TRIML1*. However, unlike eutherian genomes, which have exactly one gene annotated as *TRIML1* per species, multiple probable *TRIML1* genes are annotated in each of the four available marsupial genomes and the monotreme platypus genome. This result is possibly due to genome assembly limitations and gene annotation errors intrinsic to draft genome assemblies (Denton et al. 2014) and exacerbated by the similarities between members of the large *TRIM* family that complicate the differentiation between orthologs and paralogs (Tekaia 2016). We have used RNA-seq data to complement gene annotation information, knowing that human *TRIML1* and *TRIML2* show restricted tissue distribution that is biased toward testis and placenta (fig. 2, see next section for detailed verification). As in the platypus genome only one of the annotated *TRIML1* transcripts (*XM_007670587*) appears restricted in expression in ovary and low-level expression in testis (GSE43520, Necsulea et al. 2014; GSE97367, Marin et al. 2017), assuming that the expression pattern of *TRIML1* gene is somewhat conserved, we suggest that this sequence is the ortholog of eutherian *TRIML1*. Of the four marsupial species with genomes curated by NCBI, the gray short-tailed opossum is the best studied. Two probable *TRIML1* gene transcripts (*XM_007491153* and *XM_016422056*) are supported by RNA-seq data with restricted expression in testis and ovary (GSE43520, Necsulea et al. 2014; GSE97367, Marin et al. 2017; GSE106077, Chen et al. 2019) and low-level expression in placenta (GSE45211, Wang et al. 2014; GSE79121, Armstrong et al. 2017). Using the corresponding protein sequences as queries to search against the other three marsupial protein databases, we found high-level homology (bit-score >500, e-value 0.0) to exactly one probable TRIML1 protein per species (*Vombatus ursinus* XP_027712198, *Phascolarctos cinereus* XP_020831575, and *Sarcophilus harrisii* XP_012400891). Interestingly, of all potential marsupial TRIML1 proteins, only these three TRIML1 proteins and the opossum TRIML1 XP_ 007491215 (protein product of *XM_007491153*) meet the Reciprocal Best Hits criterion with each other, supporting the idea that they form a single marsupial ortholog group. As three of the four marsupial genomes have only one copy of this gene, and genome fragmentation during sequencing tends to falsely increase predicted gene numbers (Denton et al. 2014), the two highly similar opossum *TRIML1* genes (82% identical for mRNA and 71% identical for protein) located in tandem are more likely to be genome assembly and annotation artifacts, although it cannot be ruled out that a unique gene duplication event happened in opossum lineage. We constructed phylogenetic trees of *TRIML1* and *TRIML2* based on mRNA and protein sequences, using *Gekko japonicus TRIM39-like*, the reptilian *TRIM* that shows the highest homology to both *TRIML1* and *TRIML2*, as outgroup. Using both *G. japonicus TRIM39-like* and mammalian *TRIM39* sequences as outgroups does not change the topology of the part of the phylogenetic tree concerning the evolutionary relationship of *TRIML1* and *TRIML2*, thus supporting the choice of *TRIM39* as an outgroup (supplementary fig. S2, Supplementary Material online). Using both potential opossum *TRIML1* sequences or only one does not change the overall tree topology regarding eutherian *TRIML1*, marsupial *TRIML1* and *TRIML2* (fig. 1*D–G* and supplementary fig. S3, Supplementary Material online). Maximum likelihood trees based on mRNA (fig. 1D and supplementary fig. S3*A* and *E*, Supplementary Material online) and protein (fig. 1F and supplementary fig. S3*C* and *G*, Supplementary Material online) and Bayesian analyses based on protein (fig. 1G and supplementary fig. S3*D* and *H*, Supplementary Material online) support that *TRIML2* arose from a gene duplication event that occurred in stem eutherian lineage that also gave rise to eutherian *TRIML1.* However in these trees the alternative situation that *TRIML2* arose from a gene duplication event in the eutherian and marsupial common ancestor, followed by loss of *TRIML2* in marsupial lineage, as is supported by Bayesian analyses based on mRNA (fig. 1* * and supplementary fig. S3*B* and *F*, Supplementary Material online), cannot be ruled out with the currently available information. Nevertheless, a comparison of protein structures reveals that monotreme, marsupial, and eutherian TRIML1 all have the RING domain in the N-terminal motif, while TRIML2 lost this critical domain for E3 ligase function (fig. 5*A*). Thus the existence of the RING-less TRIML2 is eutherian-specific either through eutherian-specific genetic innovation or through eutherian-specific gene fixation.

### *TRIML1* and *TRIML2* Expression Domains Are Restricted to Preimplantation Embryo, Trophoblast, Ovary, and Testis

To test the hypothesis that TRIML1 and/or TRIML2 have functions at the maternal-fetal interface, we first assessed their expression pattern using tissue-specific RNA-Seq data from the Human Protein Atlas (Fagerberg et al. 2014). Unlike most TRIM genes, which are broadly expressed (Ozato et al. 2008), most human tissues do not express *TRIML1* or *TRIML2*, except for testis and placenta. (fig. 2*A*). *TRIM42* also exhibits testis-restricted expression, however, it is not expressed in human or mouse placenta (Fagerberg et al. 2014; Yue et al. 2014). It also lacks expression in human choriocarcinoma cell lines such as BeWo (Fagerberg et al. 2014), which does express both *TRIML1* and *TRIML2* (supplementary table S1, Supplementary Material online). Furthermore, *TRIM42* exists in reptile and bird genomes (data not shown), suggesting it is unlikely that its main role in eutherians would be in placenta.

We verified that *TRIML1* and *TRIML2* are expressed in human extraembryonic tissues at term by qPCR, and discovered that both of them show higher expression in the fetal membranes than in the placental disc, with *TRIML1* more abundant in amnion, a structure derived from the inner cell mass, and *TRIML2* more abundant in chorion, a structure with a large contribution from trophoblast (Luckett 1975; Pijnenborg et al. 1981) (one-way ANOVA, Tukey’s HSD; amnion vs. chorion, *P* < 0.01) (fig. 2*B*).

To gain detailed information on the expression pattern of *Triml1* and *Triml2* in tissues of earlier stages during eutherian pregnancy, we examined their mRNA levels by qPCR in mouse. Both genes are expressed in preimplantation embryos. However, as the embryo develops further, robust expression can no longer be detected in various fetal tissues, but is restricted to the extraembryonic structures. Despite the differences in extraembryonic structure development between mouse and human, mouse fetal membranes also show the highest expression level among extraembryonic structures, followed by the placental labyrinth, while junctional zone together with the decidua show more modest, yet robust expression (fig. 2*C*). RNAscope assays show similar expression patterns in these structures (fig. 2*E*). We tested expression at different time during pregnancy, and fetal membranes always show higher expression for both *Triml1* and *Triml2* than the labyrinth (*t*-test, *Triml1*: *P* < 0.01 for at all time points, *Triml2*: *P* < 0.05 for at all time points) (fig. 2*D*).

Mouse adult tissues confirm restricted expression patterns for both *Triml1* and *Triml2*. Although highest expression of *Triml1* is seen in adult testis, *Triml2* expression in adult tissues is lower than preimplantation embryos, placental labyrinth, and fetal membranes (fig. 2*C*). We investigated expression in the female reproductive systems in virgins and during pregnancy (E15.5); both *Triml1* and *Triml2* expression in ovary are lower than in testis and the expression of either gene was not detected in the nonpregnant uterus, nor in regions of pregnant uterus away from the implantation sites (fig. 2*C*). Thus in both human and mouse, *TRIML1* and *TRIML2* show restricted expression—yet with significant differences, as *TRIML1* shows highest expression in adult testis, while *TRIML2* is preferentially expressed in preimplantation embryos and in trophoblast-derived structures.

### *TRIML1* and *TRIML2* Expression Are Differentially Regulated during Trophoblast Differentiation

**Fig. 3.**
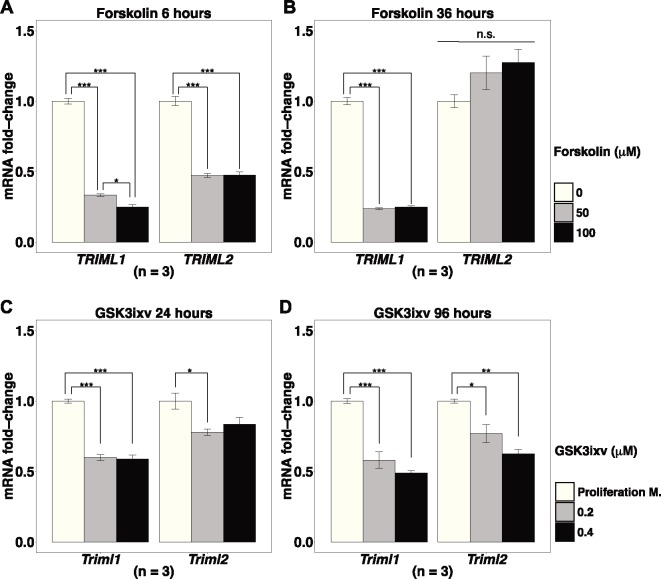
*TRIML1* and *TRIML2* expression during trophoblast differentiation. (*A* and *B*) *TRIML1* and *TRIML2* expression differ during forskolin-induced BeWo cell syncytialization. (*C* and *D*) GSK3ixv-induced mouse TSCs differentiation into syncytiotrophoblast layer II causes downregulation of both *Triml1* and *Triml2*. Expression levels normalized to control groups. Values are mean ± SEM. One-way ANOVA, Tukey’s HSD, ***P < 0.001, **P < 0.01, *P < 0.05. n.s., not statistically significant.

The differences between tissue distribution of *TRIML1* and *TRIML2* suggest that they may have acquired different cis-regulatory elements that can facilitate the acquisition of novel functions (Zhang and Zhou 2019). Although *TRIML1* and *TRIML2* reside in the same gene cluster, their head-to-head arrangement allows direction-sensitive cis-regulatory elements to influence their expression differently (Ibrahim et al. 2018; Mikhaylichenko et al. 2018). Indeed, the preferred compartment of expression within human fetal membrane appears to correlate with the direction of gene transcription, as *TRIML2*, which locates on the negative strand, shows highest expression in chorion, while the genes on the positive stand, *TRIML1* and *ZFP42* (the third gene in this gene cluster), show highest expression in amnion (figs. 1*A* and 2*B*), supporting the existence of direction-sensitive cis-regulatory elements. Therefore, we tested the hypothesis that *TRIML1* and *TRIML2* expression are differentially regulated, by examining their expression dynamics during trophoblast differentiation. Forskolin can induce BeWo choriocarcinoma cells to undergo cell fusion in a process similar to placenta syncytiotrophoblast formation (Muroi et al. 2009). Both *TRIML1* and *TRIML2* are expressed in BeWo cells (supplementary fig. S5 and table S1, Supplementary Material online). The mRNA abundance of both *TRIML1* and *TRIML2* after 6 h of forskolin treatment show significant downregulation compared with the control groups treated only with the solvent DMSO (one-way ANOVA, Tukey’s HSD, DMSO vs. 50 μM forskolin *P* < 0.001, DMSO vs. 100 μM forskolin *P* < 0.001) (fig. 3*A*). After 36 h of forskolin treatment, *TRIML1* is still significantly downregulated in all treated groups. However, no statistically significant differences of *TRIML2* remain between forskolin-treated groups and DMSO control groups, suggesting the regulation of *TRIML2* expression is highly time-sensitive (one-way ANOVA, Tukey’s HSD; *TRIML1*: DMSO vs. 50 μM forskolin *P* < 0.001, DMSO vs. 100 μM forskolin *P* < 0.001; *TRIML2*: *P* = 0.51) (fig. 3*B*).

One caveat of using BeWo cells is that the expression pattern changes observed in carcinoma cell lines may reflect the process specific to malignancy rather than normal trophoblast differentiation. Therefore, we verified the results in mouse trophoblast stem cells (TSCs), which can be differentiated to syncytiotrophoblast layer II (SynT-II) in vitro by canonical Wnt pathway activation, using specific GSK3β inhibitor, GSK3ixv (Zhu et al. 2017). GSK3ixv treatment significantly downregulated both *Triml1* and *Triml2* at 24 h compared with TSCs cultured with proliferation media and DMSO solvent (one-way ANOVA, Tukey’s HSD; *Triml1*: proliferation media vs. 0.2 μM GSK3ixv *P* < 0.001, proliferation media vs. 0.4 μM GSK3ixv *P* < 0.001; *Triml2*: proliferation media vs. 0.2 μM GSK3ixv, *P* < 0.05) (fig. 3*C*). This downregulation persisted at 96 h for both *Triml1* and *Triml2* (one-way ANOVA, Tukey’s HSD; *Triml1*: proliferation media vs. 0.2 μM GSK3ixv *P* < 0.001, proliferation media vs. 0.4 μM GSK3ixv *P* < 0.001; *Triml2*: proliferation media vs. 0.2 μM GSK3ixv *P* < 0.05, proliferation media vs. 0.4 μM GSK3ixv *P* < 0.01) (fig. 3*D*). Thus in both human choriocarcinoma BeWo cell line and in mouse TSCs, syncytialization reduces expression of *TRIML1*, which is consistent with the low *TRIML1* expression level observed in term human placenta. Although *TRIML2* did not show sustained downregulation in BeWo, its expression is significantly reduced as mouse TSCs differentiate to SynT-II cells. This difference may reflect the difference between the anatomy of mouse and human interhemal membranes. The mouse interhemal membrane consists of two layers of SynTs, while the human interhemal membrane consists of only one layer of SynTs (Coan et al. 2005). Syncytin b, the fusogenic protein in mouse SynT-II is closely related to syncytin-2 in human cytotrophoblasts, the less differentiated trophoblasts that continuously fuse to syncytiotrophoblasts (Imakawa and Nakagawa 2017). The downregulation of *Triml2* observed in mouse SynT-II may correspond to the transient *TRIML2* downregulation during BeWo cell fusion (fig. 3*A*). Thus, *TRIML1* and *TRIML2* may be regulated differently during trophoblast differentiation, especially toward the final stage of syncytialization.

### *TRIML2* Attenuates Trophoblast Proinflammatory Cytokine Production and Promotes Trophoblast Survival

**Fig. 4.**
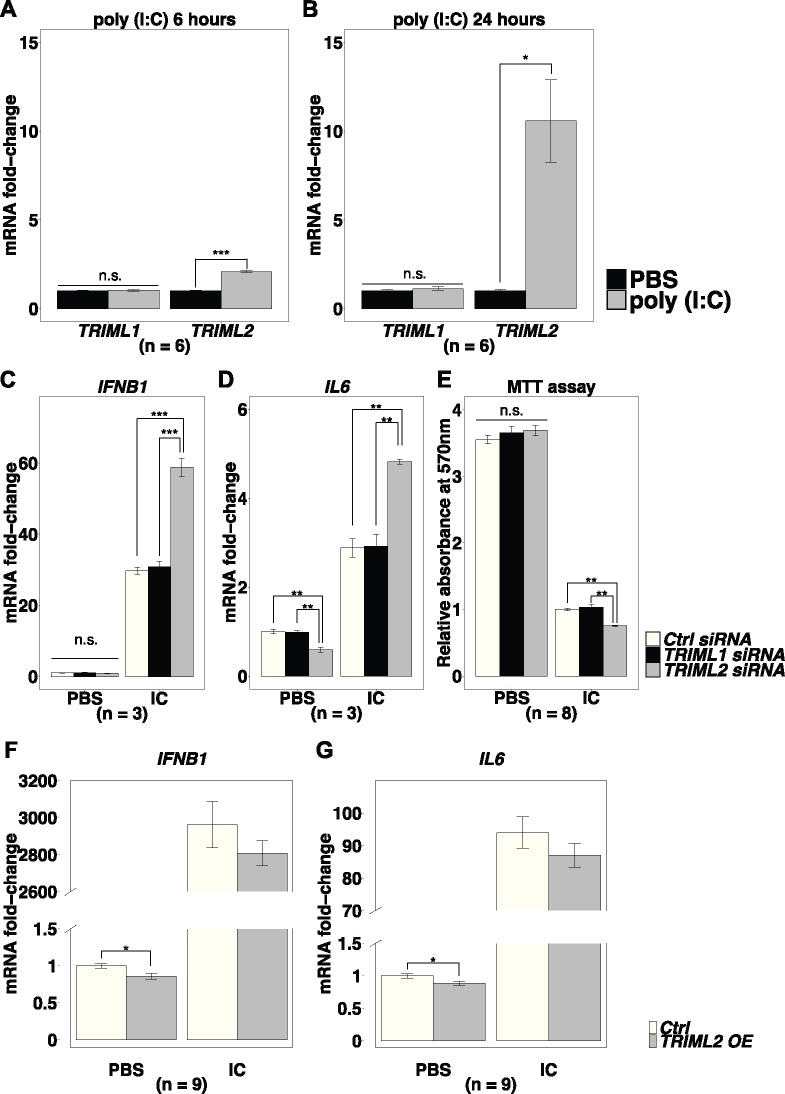
*TRIML2* during trophoblast inflammation. (*A* and *B*) *TRIML2* expression in JEG3 is induced by poly(I:C). (*C–E*) *TRIML2* knockdown during inflammation causes increased expression of proinflammatory cytokines *IFNB1* (*C*) and *IL6* (*D*), and decreased survival of JEG3 cells (*E*). *TRIML2* overexpression reduces *IFNB1* (*F*) and *IL6* (*G*) expression at baseline. Normalized to PBS-treated control groups; IC, poly(I:C), OE, overexpression. Values are mean ± SEM. (*A*, *B*, *F*, and *G*), *t*-test. (*C* and *D*) One-way ANOVA, Tukey’s HSD. Poly(I:C) treatment in (*E*), Kruskal–Wallis test, Bonferroni post hoc correction. ***P < 0.001, **P < 0.01, *P < 0.05. n.s., not statistically significant.

As *TRIML1* and *TRIML2* may be differentially regulated during trophoblast differentiation, we tested whether their expression responds differently to activation of proinflammatory pathways in trophoblasts. Previous studies have shown that many TRIMs can be induced during antiviral immune responses (Carthagena et al. 2009). Viruses are a driving force involved in both TRIM gene family expansion, as well as the evolution of eutherian placentation (Si et al. 2006; Imakawa and Nakagawa 2017). We used poly(I:C), a synthetic analog of dsRNA, to simulate viral infection associated proinflammatory pathway activation. Since transfection reagent alone causes downregulation of both *TRIML1* and *TRIML2* in BeWo cells (data not shown), we used JEG3 choriocarcinoma cells, which also express both *TRIML1* and *TRIML2* (supplementary fig. S5 and table S1, Supplementary Material online), to test this hypothesis. Poly(I:C) transfection in JEG3 induced 2-fold upregulation of *TRIML2* at 6 h (*t*-test, *P* < 0.001) and 12-fold upregulation at 24 h (*t*-test, *P* < 0.05), compared with control groups treated only with transfection reagent. *TRIML1* did not show significant response to poly(I:C) treatment (fig. 4*A* and *B*).

We then tested *TRIML1* and *TRIML2*’s influence on the expression of cytokines. Using gene targeting siRNAs, we achieved 78.4% ± 3.3% (in control groups treated with transfection reagent alone) and 79.4% ± 4.0% (in groups transfected with poly(I:C)) reduction of *TRIML1* mRNA and 82.4% ± 0.4% (in control groups treated with transfection reagent alone) and 69.3% ± 4.0% (in groups transfected with poly(I:C)) reduction of *TRIML2* mRNA, compared with control siRNA-treated groups (*n* = 3, mean ± SD). At baseline, neither *TRIML1* nor *TRIML2* siRNA treatment caused any significant differences in *interferon beta 1* (*IFNB1*) expression compared with control siRNA-treated groups (one-way ANOVA, *P* = 0.373). Upon poly(I:C) treatment however, siRNA knockdown of *TRIML2* caused 2-fold upregulation in *IFNB1* (one-way ANOVA, Tukey’s HSD, *TRIML2* siRNA vs. control siRNA, *P* < 0.001) (fig. 4*C*).

*IL6* is upregulated in preimplantation embryo during the implantation window (Basak et al. 2002). However, elevated serum IL6 is associated with spontaneous abortion in both human and mouse (Zenclussen et al. 2000, 2003). *TRIML2* knockdown significantly decreased baseline *IL6* expression in JEG3 (one-way ANOVA, Tukey’s HSD, *TRIML2* siRNA vs. control siRNA, *P* < 0.01). But upon poly(I:C) treatment, *TRIML2* knockdown resulted in 1.7-fold upregulation of *IL6* (one-way ANOVA, Tukey’s HSD, *TRIML2* siRNA vs. control siRNA, *P* < 0.01) (fig. 4*D*).

Although proinflammatory cytokines produced by trophoblasts may contribute to implantation, excessive inflammation in trophoblasts causes cell death, and is associated with pregnancy loss (Johnson et al. 1993; Aldo et al. 2010). At baseline, no difference in cell survival was observed among *TRIML1*, *TRIML2*, and control siRNA-treated groups (one-way ANOVA, *P* = 0.46). However, upon poly(I:C) transfection, *TRIML2* knockdown significantly reduced JEG3 survival (Kruskal–Wallis test, Bonferroni post hoc correction, *TRIML2* siRNA vs. control siRNA, *P* < 0.01) (fig. 4*E*).

As reduced cell survival with *TRIML2* knockdown is observed, especially during poly(I:C) transfection, it is possible that the increased proinflammatory cytokine level reflected the generally compromised cell function and the breakdown of homeostasis. To test whether *TRIML2* is directly involved in proinflammatory pathway regulation, we transfected JEG3 with a human *TRIML2* expression plasmid and achieved a 3.7 ± 0.2-fold increase of *TRIML2* mRNA at baseline, and 11.3 ± 0.6-fold increase during poly(I:C) transfection compared with control groups transfected with the corresponding vector plasmid (*n* = 9, mean ± SD). Due to the highly efficient vector transfection, *TRIML2* overexpression was achieved with reduced dosage of the transfection reagent, which significantly decreased cell death and improved cell function as reflected by the higher fold increment in the proinflammatory cytokine production upon poly(I:C) stimulation compared with siRNA-treated groups. At baseline, both *IFNB1* and *IL6* were significantly decreased by *TRIML2* overexpression (*t*-test, *P* < 0.05). During poly(I:C) transfection, both *IFNB1* and *IL6* show trends of downregulation in *TRIML2* overexpressed groups, however, the differences are not statistically significant (*t*-test; *IFNB1*: *P* = 0.097; *IL6*: *P* = 0.457) (fig. 4*F* and *G*). *TRIML2* overexpression during poly(I:C) transfection also increased the survival of JEG3 cells by 2.3% (*n* = 9, *t*-test, *P* = 0.039). These results lend further support to the hypothesis that *TRIML2* is specifically regulated, participates in the attenuation of proinflammatory pathway in trophoblast, and promotes trophoblast survival, especially during viral infection.

### PRY/SPRY Domain of TRIML2 Shows Signature of Significant Positive Selection

**Fig. 5.**
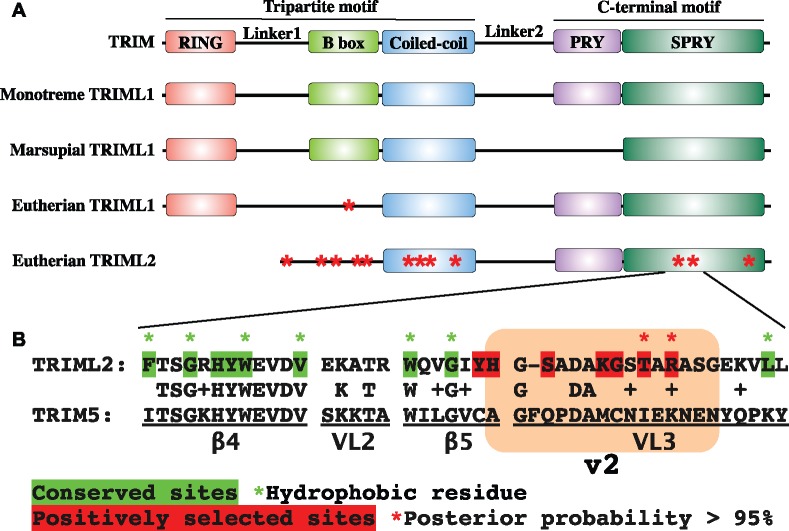
Sites under positive selection in *TRIML1* and *TRIML2*. (*A*) Compared with monotreme and marsupial TRIML1, eutherian TRIML1 lacks the B-box domain, while TRIML2 lacks both the RING and the B-box domains. In TRIML1, the site under significant positive selection resides in B-box homologous region. In TRIML2, sites under significant positive selection concentrate in B-box homologous region, coiled-coil domain and PRY/SPRY domain. (*B*) Alignment of human TRIML2 and TRIM5. Positively selected sites (red) cluster in the specificity-determining v2 region (orange shade) and are flanked by conserved sites (green) that contribute to the hydrophobic core of TRIM’s canonical binding interface.

The function of TRIMs closely correlates to their structure. We searched the conserved domains of TRIML1 and TRIML2 by RPS-BLAST (Marchler-Bauer et al. 2015) and COILS (Lupas et al. 1991), and found that, compared with their monotreme and marsupial homologs, eutherian TRIML1 lack the B-box domain, while TRIML2 lack both the B-box and the RING domains (fig. 5*A*). As TRIML2 lack both the RING and B-box domains that may adopt RING-like folds (as in the case of the RING-less TRIM16; Bell et al. 2012), it is unlikely that TRIML2 participates in the downregulation of proinflammatory pathway by acting as an E3 ubiquitin ligase. Previous studies show that TRIMs can form homo/hetero oligomers through the coiled-coil domain, and this process can dramatically influence their catalytic function (Sanchez et al. 2014). It is possible that TRIML2 interacts with other TRIMs via its coiled-coil domain, and, by influencing the E3 function of other classical TRIMs, indirectly regulates the activity of proinflammatory pathways, such as the interferon pathway. Thus TRIML2 may fine-tune the activity of ubiquitination signaling pathway without preserving its own enzymatic function. We suggest this may relax the selective constraint on *TRIML2*. To test the hypothesis of relaxed constraint, we modeled the d*N*/d*S* ratio (ω) of the homologous regions of eutherian *TRIML1* and *TRIML2* based on the phylogenetic tree generated through TimeTree website (Kumar et al. 2017; supplementary fig. S1, Supplementary Material online). Comparing the fit of one-ratio model (M0), which assigns the same ω to both *TRIML1* and *TRIML2*, with the fit of a two-ratio model (M2), which allows ω parameters to differ between *TRIML1* and *TRIML2*, M2 gives a significantly better fit than M0, with *TRIML2* ω (0.437) significantly higher than that of *TRIML1* (0.168), supporting a relaxed selection constraint on *TRIML2* (table 1).

**Table 1.** ω and Likelihood Ratio Test (LRT) of Eutherian *TRIML1* and *TRIML2*.

Gene Region	Model	Parameters	lnL^a^	*P* Value
Homologous region of *TRIML1* and *TRIML2*	M0	ω = 0.2983	−29,277.6	4.58e-65
	M2	ω*T1* = 0.168 ω*T2* = 0.437	−29,132.5	
Homologous region of *TRIML1*	M1a	*p*1 = 0.8288, ω1 = 0.095 *p*2 = 0.1712, ω2 = 1.000	−17,649.8	1.00
	M2a	*p*1 = 0.8288, ω1 = 0.095 *p*2 = 0.1712, ω2 = 1.000 *p*3 = 0.0000, ω3 = 1.933	−17,649.8	
	M7β	*p* = 0.3580, *q* = 1.6188	−17,483.4	0.008
	M8β&ω	*p*0 = 0.9785, *p* = 0.4073 *q* = 2.2013, (*p*1 = 0.0215) ω = 1.094	−17,478.6	
Homologous region of *TRIML2*	M1a	*p*1 = 0.5441, ω1 = 0.234 *p*2 = 0.4559, ω2 = 1.000	−24,785.1	1.14e-8
	M2a	*p*1 = 0.5254, ω1 = 0.237 *p*2 = 0.4285, ω2 = 1.000 *p*3 = 0.0460, ω3 = 1.956	−24,766.8	
	M7β	*p* = 0.7472, *q* = 0.7273	−24,821.7	9.63e-16
	M8β&ω	*p*0 = 0.8591, *p* = 1.0102 *q* = 1.3469, (*p*1 = 0.1409) ω = 1.438	−24,787.1	
*TRIML1 PRY/SPRY*	M0	ω = 0.083	−5,936.0	
	M2	ωLab = 0.089, ωVil = 0.074	−5,935.3	0.236
	M2	ωHemo = 0.077 ωEndo = 0.080 ωEpi = 0.111	−5,934.2	0.161
*TRIML2 PRY/SPRY*	M0	ω = 0.421	−9,476.6	
	M2	ωLab = 0.451 ωVil = 0.365	−9,474.5	0.039
	M2	ωHemo = 0.439 ωEndo = 0.370 ωEpi = 0.407	−9,475.8	0.419

lnL, log likelihood values; ω*T1*, ω of *TRIML1* branches; ω*T2*, ω of *TRIML2* branches; ωLab, ω of labyrinthine and lamellar branches; ωVil, ω of villous and trabecular branches; ωHemo, ω of hemochorial branches; ωEndo, ω of endotheliochorial branches; ωEpi, ω of epitheliochorial branches.

As genes under relaxed selection constraint have been shown to contribute to evolutionary diversification (Hunt et al. 2011), we hypothesized that some sites of *TRIML2* may show evidence of adaptive evolution. We used two sets of site models (comparison between M1a nearly neutral and M2a positive selection, and comparison between M7β and M8β&ω) to test the hypothesis, and then used Bayes empirical Bayes (BEB) approach implemented in models M2a and M8β&ω to identify sites evolving under positive selection. Both M2a and M8β&ω fit the data for *TRIML2* significantly better than their corresponding neutral selection models (table 1). BEB detected twelve sites with posterior probabilities for ω > 1 greater than 95%. Ten of the twelve sites are located within the region homologous to the monotreme and marsupial TRIMLs’ B-box domain, the hinge region of the coiled-coil domain and the PRY/SPRY domain (fig. 5*A* and supplementary table S2, Supplementary Material online). Interestingly, the only site under significant positive selection in *TRIML1* (122 A in human ortholog) resides within the region that is homologous to the B-box domain of monotreme and marsupial TRIMLs. Two additional sites within the region (114 A and 126 H) also suggest positive selection, although the posterior probabilities of these two sites having ω  > 1 do not reach statistical significance (supplementary table S2, Supplementary Material online). The substitution by alanine at residue 114 altered the last cysteine in the CHC3H2 Zinc finger in the B-box domain, suggesting that the loss of B-box in eutherian TRIMLs is under positive selection.

Another domain in TRIML2 with multiple positively selected sites is the PRY/SPRY domain. Previous studies have resolved the protein structure of the PRY/SPRY domains of several TRIMs including the retrovirus-restricting TRIM5, and identified a canonical binding interface. β-sheets form the conserved hydrophobic core of this canonical binding interface. Variable loops (VLs), which show great variation among TRIMs, connect these conserved β-sheets. Among these VLs, the N-terminal of VL3 forms the variable region 2 (v2) with the last C-terminal residue of β5 immediately in front. V2 is known to contribute to the determination of retrovirus restriction specificity of TRIM5 (James et al. 2007). Alignment of human TRIML2 and TRIM5 revealed that two of the three PRY/SPRY sites under significant positive selection and four additional residues that also show evidence of positive selection all reside within v2, with β4, one of the most conserved regions in eutherian TRIML2, in vicinity (fig. 5*B*). By analogy, these results suggest that the selection in TRIML2 may act on its ability to interact with viruses. As integration of genes and/or regulatory elements originating from retroviruses has made significant contributions to the evolution and diversification of eutherian placentation (Chuong et al. 2013; Imakawa and Nakagawa 2017), by influencing defense against retroviruses, *TRIML2* may participate in the selection of retroviruses that become incorporated into the host genome, and through coevolution with retroviruses, indirectly influence the evolution and diversification of eutherian placentation. We tested the hypothesis that the PRY/SPRY domain is under different selection among species with different placentation traits by comparing the one-ratio model (M0) to the two-ratio/three-ratio models (M2), which allow branches with different placental interdigitation/invasiveness to have different ω parameters (see supplementary fig. S1, Supplementary Material online, for phylogenetic tree generated through TimeTree and the placentation type of the species). For *TRIML1*, no significant improvement was observed with M2 models. But for *TRIML2*, the M2 model that allows two different ω parameters corresponding to different degrees of placental interdigitation is a significantly better fit than M0. It suggests higher ω (0.451) on branches with high degrees of interdigitation (labyrinthine or lamellar) and lower ω (0.365) on branches with low degrees of interdigitation (villous or trabecular) (table 1), supporting that selective pressure on *TRIML2* may vary with placental interdigitation.

## Discussion

The roughly 5,100 extant eutherian mammal species with vastly different life histories testify to the success of Eutherian evolution (O’Leary et al. 2013). The innovation that separates eutherian implantation process and marsupial attachment reaction is the eutherian-specific anti-inflammatory phase that prevents the premature onset of parturition (Chavan et al. 2017; Griffith et al. 2017). Indeed, the characteristic pathways regulating implantation are conserved and modified from ancestral inflammation reactions to embryo attachment, a reaction shared with marsupials (Griffith et al. 2017). On the other hand, eutherian placenta, clearly also critical for this success, is one of the most diverse organs, and probably represents many parallel evolutionary pathways (Garratt et al. 2013).

During past years, significant advances have been made in understanding of the mechanisms that modified the inflammatory pathways on the maternal side (Chaouat 2013; Chavan et al. 2016). On the fetal side, trophoblasts can secrete monocyte attractants and other signal molecules to influence the migration and cytokine production by decidual monocytes, thus participating in the regulation of inflammation at the maternal-fetal interface (Mor 2008). Implantation-related hormonal signals trigger upregulation of proinflammatory cytokines such as IL6 in blastocyst trophoectoderm (Basak et al. 2002) and accordingly, evidence has linked spontaneous abortions to excessive expression of proinflammatory cytokines such as IL6 in placenta (Zenclussen et al. 2003). Similar to maternal tissues, fetal tissues likely evolved anti-inflammatory mechanisms that modify the ancestral pathways activated by attachment and implantation, in order to establish a stable maternal-fetal interface and prolonged pregnancy for eutherian reproduction.

To find genetic novelties that may contribute to the anti-inflammatory mechanisms on the fetal side, we carried out comparative genomic studies and found that *TRIML2*, a noncanonical member of the TRIM family, exists in the genomes of all four extant eutherian superorders, but is absent in marsupial or monotreme genomes. Phylogenetic reconstructions of *TRIML2* and its closest homologs in mammalian genomes, that is, eutherian, marsupial, and monotreme *TRIML1*s, suggest that the most likely scenario is that a duplication event in the eutherian stem lineage gave rise to both *TRIML2* and eutherian *TRIML1* (fig. 1*D*, *F*, and *G*). However, the alternative explanation, that *TRIML2* arose from a gene duplication event in the eutherian and marsupial common ancestor, followed by eutherian-specific gene fixation and marsupial-specific gene loss, cannot be entirely ruled out (fig. 1E). Some degree of caution should be taken with the phylogenetic reconstruction due to the scarcity of marsupial and monotreme genomes and potential genome assembly and/or gene annotation errors associated with draft genomes. However, despite the uncertainty regarding the evolutionary origin of the RING-less TRIML2, its absence in marsupial genomes and ubiquitous preservation in eutherian genomes still indicates that it contributes to the evolutionary divergence of eutherian and marsupial.

Duplicated genes that are preserved in the genome may go through either neofunctionalization or subfunctionalization. A common signature of gaining new functions is the establishment of new expression pattern via recruitment of new cis-regulatory elements (Zhang and Zhou 2019). Although both *TRIML1* and *TRIML2* show highly restricted tissue distribution, they differ in their specific expression compartments. The highest expression of *TRIML1* is in adult testis, followed by preimplantation embryos and extraembryonic tissues derived from inner cell mass, such as amnion. *TRIML2* shows higher expression in fetal than adult tissues, and in extraembryonic tissues it shows higher expression in trophoblast-derived tissues, such as chorion (fig. 2). The divergence in expression compartments of the *TRIML*s parallels the divergence in direction of transcription, as *TRIML1* shows greater similarity to the expression pattern of *ZFP42*, the third member in the gene cluster which transcribes in the same direction as *TRIML1* but opposite to *TRIML2* (fig. 2B). Previous studies have shown that neighboring genes can share cis-regulatory elements such as enhancers (Quintero-Cadena and Sternberg 2016). As *ZFP42* encodes REX-1, an embryonic stem cell (ESC) pluripotency marker (Scotland et al. 2009), it is possible that by sharing direction-sensitive cis-regulatory elements, such as unidirectional/skewed enhancers (Ibrahim et al. 2018; Mikhaylichenko et al. 2018), *TRIML1* establishes preferred expression in structures derived from ESCs, while the acquisition of direction-sensitive trophoblast-specific cis-regulatory elements by *TRIML2* further separated the expression patterns. Indeed, BMP4-induced differentiation of human ESCs to trophoblast significantly upregulates *TRIML2*, while *TRIML1* shows a trend toward downregulation (GSE30915, Marchand et al. 2011). Downregulation of *TRIML1* also accompanies the differentiation of BeWo cells (fig. 3A and *B*), supporting the divergence of *TRIML1* and *TRIML2* expression patterns, which may facilitate the divergence of their functions.

Although *TRIML2* is preferentially expressed by trophoblasts, its expression is constitutively higher in fetal membranes (fig. 2D), where excessive inflammation is especially detrimental to the maintenance of pregnancy (Chavan et al. 2017). *TRIML2* may be transiently downregulated at the early stage of trophoblast differentiation (fig. 3), which may reduce the interference with the generation of proinflammatory cytokines necessary for initial placental development. *TRIML2* expression is induced by activation of proinflammatory pathways in trophoblast (fig. 4A and *B*). These expression patterns fit what would be expected for genes fine-tuned for the regulation of inflammation in trophoblast.

Functionally, losing the catalytic RING domain may facilitate the preservation of *TRIML2* in the genome through subfunctionalization. Losing most of the catalytic function may also enable TRIML2 to function as a dominant inhibitor of the inflammatory pathway, as illustrated by the increased proinflammatory cytokine production and reduced trophoblast survival during its knockdown, and reduced proinflammatory cytokine production and increased cell survival during its overexpression (fig. 4). A recent study has shed light on the potential mechanisms though which *TRIML2* promotes cell survival. *TRIML2* knockdown in human oral squamous carcinoma cells upregulates p27^Kip1^, a cyclin-dependent kinase inhibitor and apoptosis inducer that can be induced by TP53 (Wang et al. 1997; Hayashi et al. 2019). Interestingly, TRIML2 can be induced by TP53 Arg72 variants, but not by Pro72 variants (Kung et al. 2015). Higher risks of recurrent implantation failure and recurrent pregnancy loss are associated with TP53 Pro72 variants when compared with Arg72 variants (Kang and Rosenwaks 2018). It is intriguing to consider whether the lack of efficient induction of TRIML2 by TP53 Pro72 variants may result in abnormally high level of p27^Kip1^, which in turn may cause excessive apoptosis of trophoblasts, thus contributing to the increased risk of adverse pregnancy events. As the establishment and maintenance of pregnancy involve complex interplay between multiple cell types of mother and fetus, animal models with modified *TP53* and/or *TRIML2* genes may provide more information for understanding the mechanisms and differentiating the maternal/fetal contributions to the increased risk.

As similar inflammatory pathways underlie both eutherian implantation and host immunity against infectious pathogens such as viruses (Mor and Cardenas 2010; Griffith et al. 2017), the anti-inflammatory function of TRIML2 that facilitates the accommodation of the embryo at the maternal-fetal interface may also relax the stringency on viral activities at such locations. A recent study shows that *TRIML2* upregulation during Kaposi’s sarcoma-associated herpes virus activation in lymphoma cell line BCBL-1 is associated with both increased virion production and prolonged host cell survival (Gu et al. 2018). Considering the striking similarities between proliferation and invasion behaviors of trophoblast and cancer cells (Ferretti et al. 2006), a certain level of tolerance to viral activities within the placenta, a transient organ, could be beneficial compared with a full-blown immune response that may interrupt the quiescence at the maternal-fetal interface. Indeed, eutherian placenta shows substantial tolerance to retroviruses/endogenous retroviruses (ERVs) (Haig 2012; Chuong 2013). Furthermore, repeated co-option of both protein-coding genes and regulatory elements originating from ERVs play a crucial role in the rapid evolution of eutherian placentation (Haig 2012; Chuong et al. 2013; Imakawa and Nakagawa 2017). Functioning as a negative regulator, the RING-less TRIML2 may contribute to establishing an accommodating environment for retroviruses/ERVs. However, as “selfish parasites,” retroviruses/ERVs can be costly to the host, therefore, host defense mechanisms that participate in the selection of retroviruses/ERVs likely experience adaptive evolution (Haig 2012). Several amino acid residues of TRIML2 show a signature of positive selection, especially in the TRIM-interacting coiled-coil domain and the substrate-binding PRY/SPRY domain (fig. 5A and supplementary table S2, Supplementary Material online). Coiled-coil domain-mediated dimerization can dramatically influence TRIMs’ catalytic function (Sanchez et al. 2014). In some retroviral-restricting TRIMs, such as TRIM5α, subsequent higher-order association can result in the formation of a hexagonal lattice that matches the lattice formed by viral capsid and significantly restrict the activities of the retroviruses (Ganser-Pornillos et al. 2011). Substituting TRIM monomers in such lattices through its coiled-coil domain, TRIML2 could interrupt the orderly structure necessary for the retroviral-restricting function. Changes in TRIML2’s PRY/SPRY domain may change its affinity to the viral capsid thus influencing the selection of retroviruses that can escape the restrictions imposed by other TRIMs.

Previous studies suggest lineage-specific ERV enhancer co-option may be the mechanism underlying the evolution of eutherian placentation diversification (Chuong et al. 2013). Corresponding to different placentation features, we observed different selection pressure of the potential virus-interacting PRY/SPRY domain of TRIML2. Significantly higher ω is associated with placentas with higher levels of interdigitation and vice versa (table 1). Higher-level interdigitation such as in labyrinthine placentation provides larger surface area for exchange between mother and fetus, and therefore can support faster fetal growth. On the other hand, rapid draining of maternal resources can exacerbate the parental-offspring-conflict (Capellini 2012; Garratt et al. 2013). Therefore, we suggest that the higher ω, associated with higher-level placental interdigitation, may reflect the intensified parental-offspring conflict, as the plasticity of *TRIML2*, resulting from relaxed selection, may facilitate the rapid modification of antiviral immune response by adjusting the expression of proinflammatory cytokines at the maternal-fetal interface. Some of these proinflammatory cytokines, such as IFNB1 (Jablonska et al. 2010) and IL6 (Gopinathan et al. 2015), likely participate in the regulation of angiogenesis, arguably resulting in rapid modification of the degree of placental interdigitation. In another prominent placentation trait, placental invasiveness, the highest ω is associated with hemochorial placentation, although the difference does not reach statistical significance (table 1). The high ω in hemochorial placentation may reflect the challenge in maintaining maternal immune tolerance in highly invasive placentas in which fetal tissue are directly exposed to maternal blood stream (Elliot and Crespi 2006).

The plasticity of TRIML2 renders high tolerance to nonsynonymous substitutions. Indeed, the Exome Aggregation Consortium (ExAC) database predicts the index of intolerance to loss of function (LoF) of *TRIML2* to be 0.00 (range 0–1, with 0 most tolerant) (Lek et al. 2016). We suggest that the modifiable nature of *TRIML2*, with tolerance even to loss of function (at least under some conditions), may increase its variation and provide a reservoir for potential coevolution with retroviruses/ERVs that may contribute to the diversification of eutherian placentation. Eutherian placenta is one of the most diverse organs despite its similar and essential core functions across species (Garratt et al. 2013). “Nonessential” immune genes, that is, genes that are only necessary when host is challenged by corresponding pathogens, are amongst the fastest evolving genes, a phenomenon likely driven by the host-parasite coevolution that favors the diversification of these immune genes (Hurst and Smith 1999). We suggest that many nonessential immune genes may participate in the coevolution with retroviruses/ERVs that results in the diversification of eutherian placentation. Through gene duplication, the TRIM family may provide many such modifiable genes, as the TRIM family shows fast expansion in the eutherian lineage and sublineages (Versteeg et al. 2014). We examined the LoF index of human TRIMs that were predicted to originate in Eutherian or sublineages (Capra et al. 2013). Out of 23 TRIMs that have records in ExAC database, the LoF index of 14 is predicted to be 0.00 (Lek et al. 2016; supplementary table S3, Supplementary Material online), including TRIMs such as TRIM5 (Ganser-Pornillos et al. 2011) and TRIM6 (Bharaj et al. 2017), that have been shown to play important roles in antiviral immunity. Through interaction with TRIMs with placentally biased expression, and/or alternative splicing that can result in the RING-less negative regulatory forms (Versteeg et al. 2013), these TRIMs may selectively permit the activity of some retroviruses/ERVs at the maternal-fetal interface, without interfering with the host’s overall antiviral immunity. Thus, the fast-evolving and versatile TRIM family may provide a wealth of evolvability in the regulation of the selective permissive placental environment to retroviruses/ERVs (Chuong 2013) and contribute to the fascinating diversification of eutherian placentation.

### Conclusion

The eutherian-specific gene *TRIML2* functions as a regulatory inhibitor of the proinflammatory cytokine production pathways in trophoblasts. We suggest that this function contributes to the establishment of the anti-inflammatory phase critical for eutherian implantation and prolonged pregnancy, and by influencing antiviral immunity against coevolving retroviruses/ERVs, also contributed to the diversification of placentation.

## Materials and Methods

### Phylogenetic Gene Tree Reconstruction and Inference of Selection

Information on placental interdigitation and invasiveness were obtained from Wildman et al. (2006), Elliot and Crespi (2009), and Garratt et al. (2013). The protein coding DNA sequences for *TRIML1* and *TRIML2* of species with known placental interdigitation and invasiveness were downloaded from NCBI Entrez Nucleotide sequences through Alignment Explorer implemented in MEGA X (Kumar et al. 2018), the integrity of the coding regions were reviewed and partial sequences were removed from analysis. SPRY domain was inferred by RPS-BLAST (Marchler-Bauer et al. 2015). Sequences were aligned using MUSCLE implemented in MEGA X. Conserved blocks from multiple alignments suitable for phylogenetic analysis were selected using Gblocks v0.91b (Castresana 2000). Phylogenetic tree was inferred by maximum likelihood with 1,000 bootstraps implemented in MEGA X and by Bayesian analyses using MrBayes v3.1.2 (Ronquist and Huelsenbeck 2003). The CodeML package implemented in PAML 4.4 was used to estimate ω (Yang 2007). All phylogenetic trees used for PAML analyses were generated through TimeTree website (Kumar et al. 2017). All selection models were compared with the neutral models using a likelihood ratio test (LRT) and null hypothesis was rejected when *P* < 0.05.

### Relative Tissue Expression Level of Human TRIM Genes

Tissue-specific RNA-Seq data for human TRIM genes was downloaded from the Human Protein Atlas, accessed through the gene portal of NCBI (last accessed October 19, 2017). For each TRIM gene, the expression level in each tissue was expressed as fraction of the amount in the tissue with highest expression level.

### Human Placenta and Fetal Membrane Samples

Three human placentas at term with attached fetal membranes were collected with IRB approval (IRB protocol: CCHMC IRB 2013–2243). Samples were washed with PBS prior to collecting three tissue samples from the villous side and three tissue samples from the decidual side of each placenta. Fetal membranes were manually separated into chorion and amnion, and six samples were collected from each part.

### Cell Lines

The mouse trophoblast stem cell line was provided by Dr S.K. Dey (Xie et al. 2012) and was cultured in DMEM/F12 medium supplemented with Activin A (R&D Systems 338-AC) (100 μg/ml), heparin (1 μg/ml), FGF4 (R&D Systems 235-F4) (25 ng/ml), 20% FBS, 2-Mercaptoethanol (100 μM), sodium pyruvate (1 mM), glutaMAX (2 mM), penicillin (100 U/ml), and streptomycin (100 μg/ml). BeWo choriocarcinoma cell line was obtained from ATCC (ATCC CCL-98) and cultured in DMEM/F12 medium supplemented with 10% FBS, glutaMAX (2 mM), penicillin (100 U/ml), and streptomycin (100 μg/ml). JEG3 choriocarcinoma cell line was obtained from ATCC (ATCC HTB-36) and cultured in DMEM medium supplemented with 10% FBS, glutaMAX (2 mM), penicillin (100 U/ml), and streptomycin (100 μg/ml). All cell lines were cultured at 37 °C and 5% CO_2_.

### Mouse TSCs Differentiation

About 1 × 10^4^ mouse TSCs were seeded per well of 6-well plate. About 24 h after seeding, medium without Activin A, heparin, and FGF4, and with 0.2 μM or 0.4 μM GSK3ixv (Millipore 361558) was added. Proliferation medium described above with the same volume of solvent DMSO was used as control. Medium was exchanged every 24 h. Total RNA was collected after 24 or 96 h.

### BeWo Cell Differentiation

About 2 × 10^5^ BeWo cells were seeded per well of 6-well plate. About 48 h after seeding, culture medium was changed, and 50 μM, or 100 μM forskolin (Sigma F3917), or the same volume of solvent DMSO were added. Medium was exchanged every 24 h. Total RNA was collected after 6 or 36 h.

### Poly(I:C) Transfection

About 1 × 10^5^ JEG3 cells were seeded per well of 6-well plate. About 48 h after seeding, culture medium was changed, and high molecular weight poly(I:C) (InvivoGen tlrl-pic) was transfected at a concentration of 2 μg/ml with 4 μl/ml Lipofectamine 3000 (Invitrogen) (Palchetti et al. 2015). PBS with 4 μl/ml Lipofectamine 3000 was added to control wells. Total RNA was collected after 6 or 24 h.

### RNA Interference with Poly(I:C) Transfection

About 2 × 10^5^ JEG3 cells were seeded per well of 6-well plate. About 48 h after seeding, culture medium was changed, and siRNA for *TRIML1* (Invitrogen Silencer Select s50635), *TRIML2* (Invitrogen Silencer Select s47568), or control siRNA (Invitrogen Silencer Select Negative Control No. 2) were transfected at 100 pmol/well with 4 μl/ml Lipofectamine 3000. About 18 h after siRNA transfection, high molecular weight poly(I:C) was transfected at a concentration of 2 μg/ml with 4 μl/ml Lipofectamine 3000. PBS with 4 μl/ml Lipofectamine 3000 was added to control wells. Total RNA was collected 6 h after poly(I:C) treatment. [3-(4,5-dimethylthiazol-2-yl)-2,5-diphenyltetrazolium bromide] (MTT) assay was carried out 12 h after poly(I:C) treatment.

### *TRIML2* Overexpression with Poly(I:C) Transfection

About 1 × 10^5^ JEG3 cells were seeded per well of 6-well plate. About 24 h after seeding, culture medium was changed, and human *TRIML2* expression plasmid (OriGene RC211281L1) or vector plasmid (OriGene PS100064) were transfected at 15 ng/well with 1.5 μl/ml Lipofectamine 3000. About 24 h after expression plasmid transfection, high molecular weight poly(I:C) was transfected at a concentration of 2 μg/ml with 4 μl/ml Lipofectamine 3000. PBS with 4 μl/ml Lipofectamine 3000 was added to control wells. Total RNA was collected 6 h after poly(I:C) treatment. MTT assay was carried out 12 h after poly(I:C) treatment.

### MTT Assay

JEG3 cells were treated as described earlier. Subsequently, cells were washed three times with 500 μl PBS/well. About 800 μl/well MTT solution at 0.5 mg/ml was added to each well, and incubated at 37 °C for 3 h. About 500 μl/well of DMSO was added and plate was wrapped in foil and shaken on an orbital shaker for 15 min. About 200 μl of the resulting solution from each well was transferred from 6-well plates to a well in 96-well plates and the absorbance was measured at 570 nm wavelength.

### BeWo and JEG3 Transcriptomes

BeWo and JEG3 cells were grown under above specified conditions until 90% confluent. Cells were harvested by scraping and the total RNA was extracted from cells using the RNeasy Mini kit (Qiagen) followed by on-column DNase I treatment. RNA quality was tested using Bioanalyzer 2100 (Agilent). Samples with RIN > 9.5 were sequenced using the Illumina HiSeq 2500, to a minimum of 30 million 50 bp paired-end reads per sample. Reads were aligned with TopHat 2.1.1 to the human Ensembl cDNA build, and subsequently counted and normalized using Cufflinks 2.2.1. Two repeats of each of the two cell lines were processed independently and the expression values averaged over transcriptomes. The data have been deposited in NCBI’s Gene Expression Omnibus and are accessible through GEO Series accession number GSE131753 (https://www.ncbi.nlm.nih.gov/geo/query/acc.cgi? acc=GSE131753).

### Animals

All studies on mice were approved by the Cincinnati Children’s Medical Center Animal Care and Use Committee (CCHMC IACUC 2017-0051) and comply with the National Institutes of Health guidelines. All studies were carried out on FVB/N background mice. Mice were housed in plastic cages with ad libitum diet and 10-h dark/14-h light cycle.

### Mouse Tissue Collection

Female FVB/N mice aged 8–10 weeks were mated with male FVB/N mice from 8 PM until 8 AM, and subsequently separated. Female mice were checked for vaginal plug (E 0.5). For each time point, tissues from three dams were collected. From E13.5 to E18.5, tissues were collected at 8 AM; for E19.0, tissues were collected at 8 PM. Fetal membranes and placenta were collected from two fetuses of each dam. The placenta was then manually separated into the labyrinth part and the junctional zone and decidual part. On E15.5, uterus and ovary were collected for each dam; fetal brain, limb, liver, and lung tissues were collected from two fetuses of each dam. Uterus and ovary tissue were also collected from three virgin female mice. Testis was collected from six males. Other adult tissues, including brain, kidney, liver, lung, muscle, and spleen were each collected from six mice (three males and three females).

For mouse preimplantation embryo collection, 3-week-old female FVB/N mice were treated with pregnant mare serum gonadotropin (Prospec PMSG) 5 IU/mouse through peritoneal injection at 12 PM. About 47 h after PMSG injection (11 AM), mice were treated with hCG (Millipore 230734) 5 IU/mouse through peritoneal injection, then mated with male FVB/N mice. Female mice were separated from male at 8 AM next morning, and vagina plug was check at that time. About 42–44 h, 68–70 h, and 98–100 h after hCG injection, 2-cell stage embryos, 8-cell stage embryos, and blastocyst were collected, respectively. Embryos from the same female were combined for total RNA collection.

### Total RNA Isolation and Quantitative PCR

Total RNA from ovary and preimplantation embryos was isolated using RNeasy plus micro kit (Qiagen). Total RNA from all other tissues and cells was isolated using EZ tissue/cell total RNA miniprep kit (EZ BioResearch). RNA was reverse transcribed to cDNA using the QuantiTect Reverse Transcriptase Kit (Qiagen). Quantitative PCR (qPCR) for human term placenta and fetal membrane samples and BeWo cell differentiation was performed using EXPRESS SYBR GreenER (ThermoFisher Scientific). All other qPCRs were performed using TaqMan Fast Advanced Master Mix and predesigned TaqMan gene expression assay (ThermoFisher Scientific). All qPCR reactions were run on Applied Biosystems StepOnePlus Real-Time PCR instrument. Species-specific *GPDH* is used as endogenous control.

### RNAscope In Situ Assay of Mouse Implantation Site

On E15.5 implantation site samples (placentas with attached fetal membranes and uterus wall) were collected as described earlier. Samples were fixed in 10% neutral buffered formalin for 24 h at room temperature, washed with PBS, dehydrated in standard ethanol series followed by embedding in paraffin. Paraffin blocks were cut into 5 μm sections using a microtome. RNAscope assay was carried out using RNAscope 2.5 HD Duplex kit (Cat No. 322430). Probe-Mm-Triml1-E4-E6-C2 (Cat No. 478441-C2) and Probe-Mm-Triml2-E5-E7 (Cat No. 473731) were used to detect the target genes. 2-plex Negative Control Probe (Cat. No. 320751) was used to evaluate nonspecific signals.

### Statistical Analysis

All statistical analysis was performed in RStudio version 1.1.383. Specific statistical tests are described earlier. 

## Supplementary Material

msz238_Supplementary_Data
