# Supplementary material for: Eutherian-Specific Gene TRIML2 Attenuates Inflammation in the Evolution of Placentation
Source: Mol Biol Evol. 2019 Oct 9;37(2):507–23. doi: 10.1093/molbev/msz238 (PMC6993854; doi:10.1093/molbev/msz238)
Supplement: msz238_Supplementary_Data [file msz238_supplementary_data.pdf]

Supplementary Materials

Table S1 RNAseq results of *TRIML1* and *TRIML2* expression in BeWo and JEG3

Cell line	*TRIML1*/FPKM	*TRIML2*/FPKM
BeWo #1	6.054	3.244
BeWo #2	6.704	3.096
JEG3 #1	3.609	12.437
JEG3 #2	4.658	10.549

Table S2 Positively selected sites in eutherian TRIML1 and TRIML2

TRIML	AA#[a]	AA	ω	SD[b]	PP[c]	Domain
**TRIML1**	94	S	1.171	0.368	0.543	**Linker1**
	114	A	1.385	0.264	0.835	**B-box homologous region**
	**122**	**A****	**1.494**	**0.063**	**0.990**	
	126	H	1.155	0.384	0.539	
	235	S	1.283	0.335	0.695	**Coiled-coils coil B**
	240	A	1.161	0.383	0.548	**Linker2**
	241	F	1.409	0.240	0.870	
**TRIML2**	6	S	1.407	0.227	0.852	**N-terminal**
	7	P	1.454	0.166	0.926	
	8	Q	1.165	0.352	0.509	
	9	L	1.375	0.267	0.814	
	10	Q	1.393	0.243	0.833	
	11	H	1.460	0.154	0.936	
	**12**	**N***	**1.493**	**0.064**	**0.988**	
	13	I	1.373	0.259	0.803	
	**18**	**Y***	**1.492**	**0.068**	**0.987**	**B-box homologous region**
	21	T	1.169	0.349	0.513	
	**26**	**T****	**1.498**	**0.037**	**0.996**	
	35	I	1.418	0.214	0.870	
	42	F	1.449	0.173	0.918	
	44	S	1.205	0.350	0.572	
	45	Q	1.287	0.316	0.680	
	49	H	1.305	0.306	0.702	
	**51**	**M***	**1.494**	**0.061**	**0.989**	
	**53**	**C***	**1.494**	**0.062**	**0.989**	
	56	Q	1.175	0.356	0.531	**Coiled-coils coil A**
	61	N	1.220	0.336	0.579	
	125	Q	1.155	0.363	0.508	**Coiled-coils hinge**
	128	Q	1.186	0.352	0.543	
	**130**	**C***	**1.479**	**0.114**	**0.965**	
	**131**	**I****	**1.499**	**0.017**	**0.999**	
	134	L	1.433	0.200	0.895	
	140	L	1.376	0.257	0.807	

141	L	1.436	0.194	0.899	
146	K	1.297	0.305	0.686	
**164**	**R****	**1.499**	**0.020**	**0.999**	
171	E	1.388	0.245	0.823	**Coiled-coils coil B**
178	A	1.328	0.294	0.737	
**189**	**K***	**1.477**	**0.116**	**0.963**	
191	I	1.184	0.347	0.535	
192	V	1.326	0.294	0.734	
201	G	1.402	0.235	0.849	**Linker2**
203	L	1.419	0.217	0.876	
208	C	1.150	0.425	0.572	
231	S	1.206	0.403	0.632	
236	Y	1.456	0.161	0.930	
237	S	1.433	0.199	0.896	
246	L	1.231	0.347	0.612	
248	H	1.233	0.340	0.606	
253	H	1.463	0.150	0.940	
267	S	1.254	0.327	0.629	
270	R	1.199	0.344	0.553	
301	G	1.296	0.312	0.692	**PRY**
346	Y	1.458	0.159	0.933	**SPRY**
347	H	1.164	0.356	0.513	
349	S	1.463	0.149	0.942	
353	K	1.370	0.264	0.800	
354	G	1.466	0.143	0.945	
**356**	**T***	**1.482**	**0.103**	**0.971**	
**358**	**R***	**1.489**	**0.082**	**0.981**	
375	W	1.430	0.202	0.891	
414	M	1.424	0.208	0.879	
**422**	**H***	**1.484**	**0.097**	**0.974**	

a. AA#: Amino acid residue numbers refer to human TRIML1/TRIML2.

b. SD: standard deviation of **ω**.

c. PP: posterior probability of **ω > 1. *: PP > 95%, **: PP > 99%.**

Table S3 ExAC index of intolerance to loss of function (LoF)

Origen	TRIM	LoF
**Eutheria**	**TRIM22**	**0.00**
	TRIM28	1.00
	**TRIM38**	**0.00**
	TRIM43	0.48
	TRIM43B	NA
	TRIM49	0.65
	**TRIM49B**	**0.00**
	**TRIM50**	**0.00**
	TRIM56	0.08
	**TRIM6**	**0.00**
	**TRIM68**	**0.00**
	TRIM73	NA
	TRIM74	0.78
	**TRIML2**	**0.00**
**Euarchontoglires**	**TRIM17**	**0.00**
	TRIM21	0.09
	**TRIM5**	**0.00**
**Catarrhini**	**TRIM4**	**0.00**
	**TRIM51**	**0.00**
	**TRIM52**	**0.00**
	**TRIM60**	**0.00**
	TRIM61	0.67
	TRIM64	NA
	TRIM64B	NA
	TRIM64C	0.01
**Homininae**	**TRIM48**	**0.00**
	TRIM77	NA
**Homo sapiens**	TRIM49C	0.16

Table S4 Primers for qPCR

Species	Gene	Forward primer	Reverse primer
Human	*GPDH*	AATCCCATCACCATCTTCCA	TGGACTCCACGACGTACTCA
	TRIML1	TCACGGGAATGAAGGAGATGC	GATCCTCCGACAACACGAGA
	TRIML2	TGCCACATAAGAGGACTCAGC	CTGCTGCCCATGTCTCAATC
	ZFP42	TGGAGCCTGTGTGAACAGAAC	ACCTCCAGGCAGTAGTGATCT

Table S5 TaqMan gene expression assays for qPCR

Species	Gene	TaqMan Gene Expression Assay ID
Human	*GPDH*	Human *GAPD* (*GAPDH*) Endogenous Control (VIC™/TAMRA™ probe, primer limited)
	TRIML1	Hs00385739_m1
	TRIML2	Hs00543074_m1
	INFB1	Hs01077958_s1
	IL6	Hs00174131_m1
Mouse	*Gpdh*	Mouse *GAPD* (*GAPDH*) Endogenous Control (VIC™/MGB probe, primer limited)
	Triml1	Mm00625004_m1
	Triml2	Mm03990484_m1

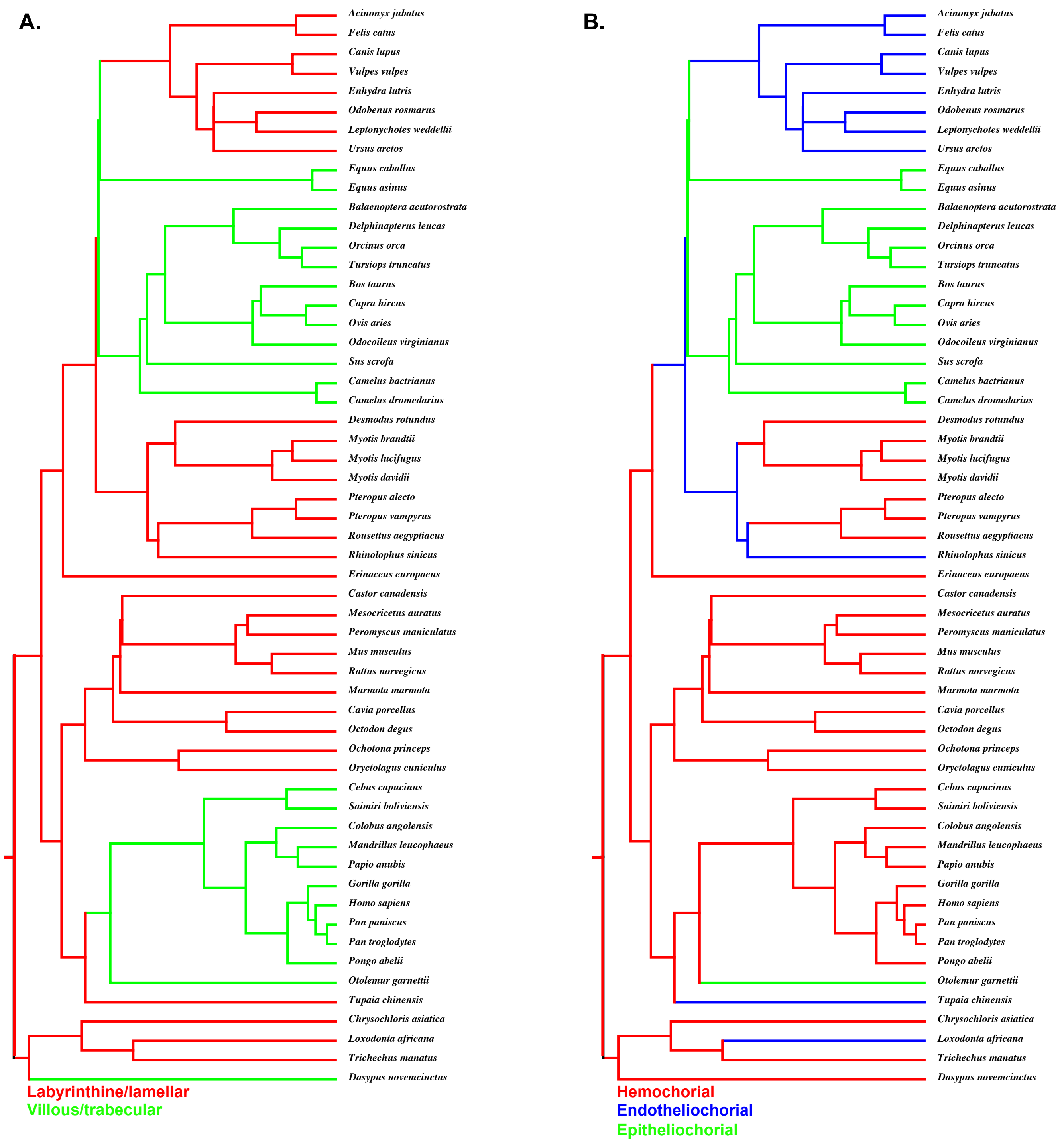

Fig S1 Placental interdigitation (A) and invasiveness (B) of species with complete *TRIML1* and *TRIML2* sequences. Phylogenetic trees were generated using Timetree.org. Data of placental traits of species listed stem from Elliot and Crespi 2009, and Garratt et al. 2013. Data of placental traits of internal nodes stem from Wildman et al. 2006, and Elliot and Crespi 2009.

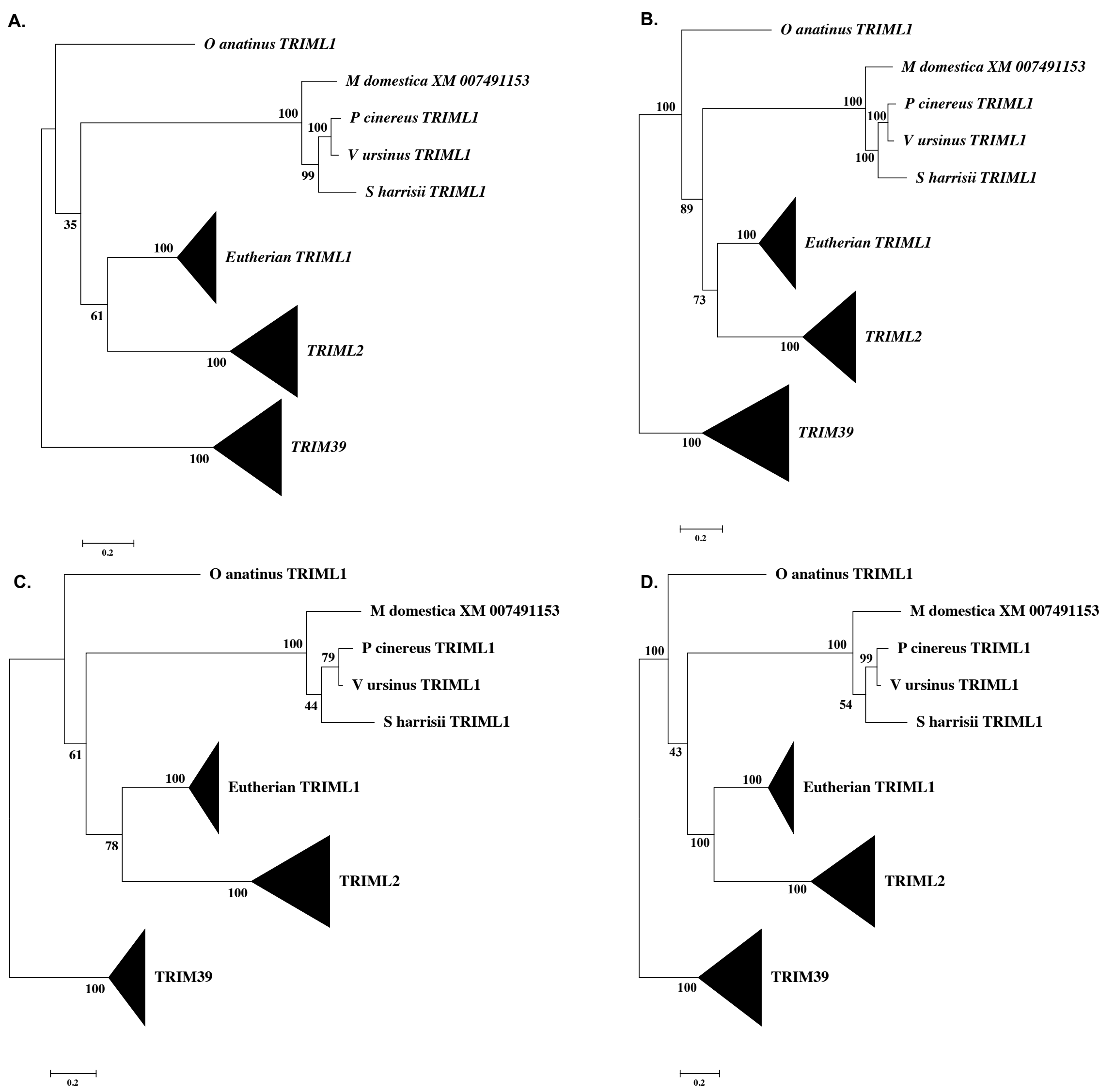

Fig S2. Phylogenetic reconstruction of *TRIML1* and *TRIML2* with *G. japonicus TRIM39-like* and mammalian *TRIM39* sequences as outgroups. Phylogenetic trees generated though maximum likelihood (A, mRNA tree; C, protein tree) and Bayesian analyses (B, mRNA tree; D, protein tree), with bootstrap percentages / posterior probabilities.

A.

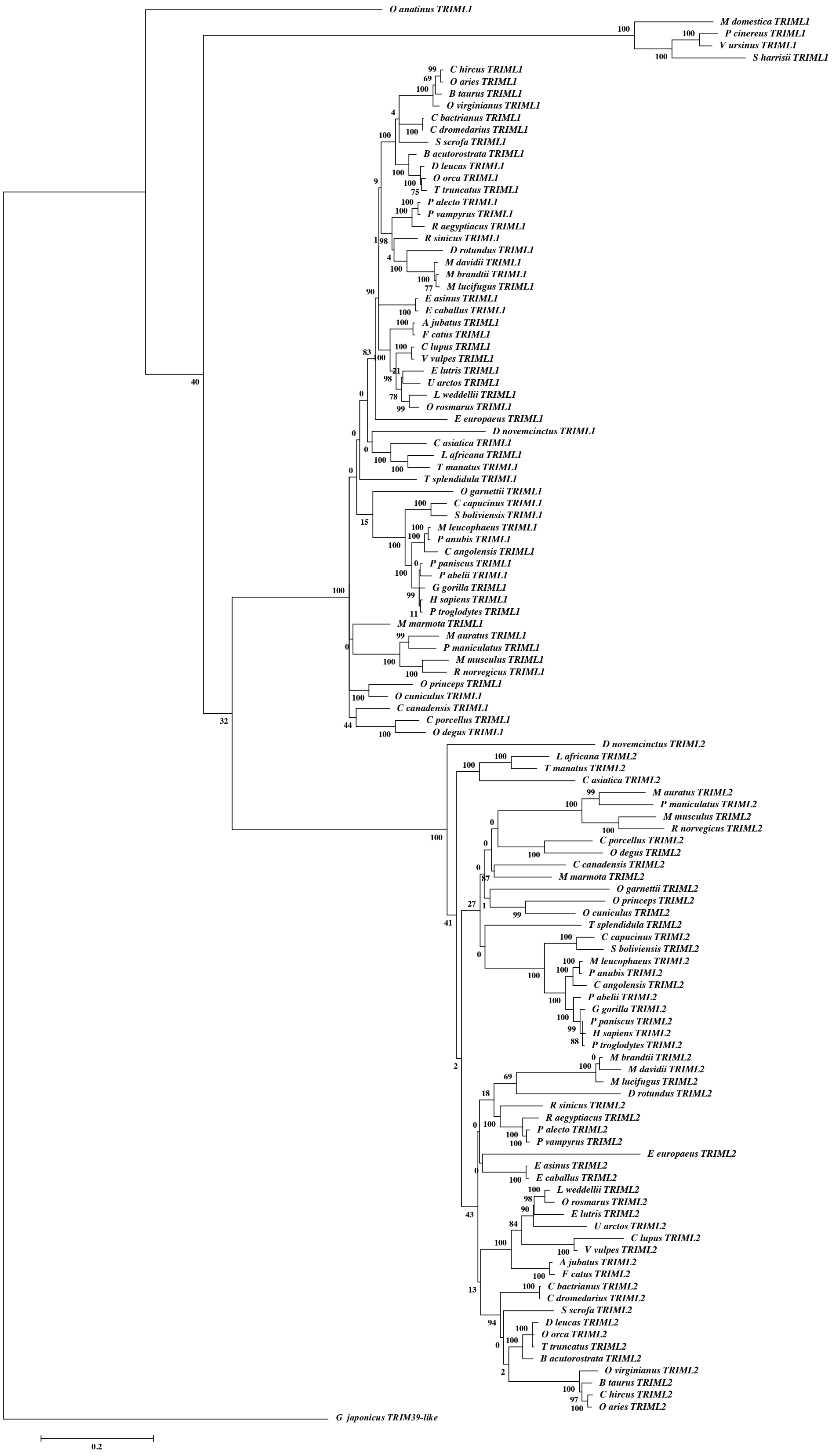

O anatinus TRIML1
M domestica TRIML1
P cinereus TRIML1
V ursinus TRIML1
S harrisii TRIML1
C hircus TRIML1
O aries TRIML1
B taurus TRIML1
O virginianus TRIML1
C bactrianus TRIML1
C dromedarius TRIML1
S scrofa TRIML1
B acutorostrata TRIML1
D leucas TRIML1
O orca TRIML1
T truncatus TRIML1
P alecto TRIML1
P vampyrus TRIML1
R aegyptiacus TRIML1
R sinicus TRIML1
D rotundus TRIML1
M davidii TRIML1
M brandtii TRIML1
M lucifugus TRIML1
E asinus TRIML1
E caballus TRIML1
A jubatus TRIML1
F catus TRIML1
C lupus TRIML1
V vulpes TRIML1
E lutris TRIML1
U arctos TRIML1
L weddellii TRIML1
O rosmarus TRIML1
E europaeus TRIML1
D novemcinctus TRIML1
C asiatica TRIML1
L africana TRIML1
T manatus TRIML1
T splendidula TRIML1
O garnettii TRIML1
C capucinus TRIML1
S boliviensis TRIML1
M leucophaeus TRIML1
P anubis TRIML1
C angolensis TRIML1
P paniscus TRIML1
P abelii TRIML1
G gorilla TRIML1
H sapiens TRIML1
P troglodytes TRIML1
M marmota TRIML1
M auratus TRIML1
P maniculatus TRIML1
M musculus TRIML1
R norvegicus TRIML1
O princeps TRIML1
O cuniculus TRIML1
C canadensis TRIML1
C porcellus TRIML1
O degus TRIML1
D novemcinctus TRIML2
L africana TRIML2
T manatus TRIML2
C asiatica TRIML2
M auratus TRIML2
P maniculatus TRIML2
M musculus TRIML2
R norvegicus TRIML2
C porcellus TRIML2
O degus TRIML2
C canadensis TRIML2
M marmota TRIML2
O garnettii TRIML2
O princeps TRIML2
O cuniculus TRIML2
T splendidula TRIML2
C capucinus TRIML2
S boliviensis TRIML2
M leucophaeus TRIML2
P anubis TRIML2
C angolensis TRIML2
P abelii TRIML2
G gorilla TRIML2
P paniscus TRIML2
H sapiens TRIML2
P troglodytes TRIML2
M brandtii TRIML2
M davidii TRIML2
M lucifugus TRIML2
D rotundus TRIML2
R sinicus TRIML2
R aegyptiacus TRIML2
P alecto TRIML2
P vampyrus TRIML2
E europaeus TRIML2
E asinus TRIML2
E caballus TRIML2
L weddellii TRIML2
O rosmarus TRIML2
E lutris TRIML2
U arctos TRIML2
C lupus TRIML2
V vulpes TRIML2
A jubatus TRIML2
F catus TRIML2
C bactrianus TRIML2
C dromedarius TRIML2
S scrofa TRIML2
D leucas TRIML2
O orca TRIML2
T truncatus TRIML2
B acutorostrata TRIML2
O virginianus TRIML2
B taurus TRIML2
C hircus TRIML2
O aries TRIML2
G japonicus TRIM39-like
0.2

B.

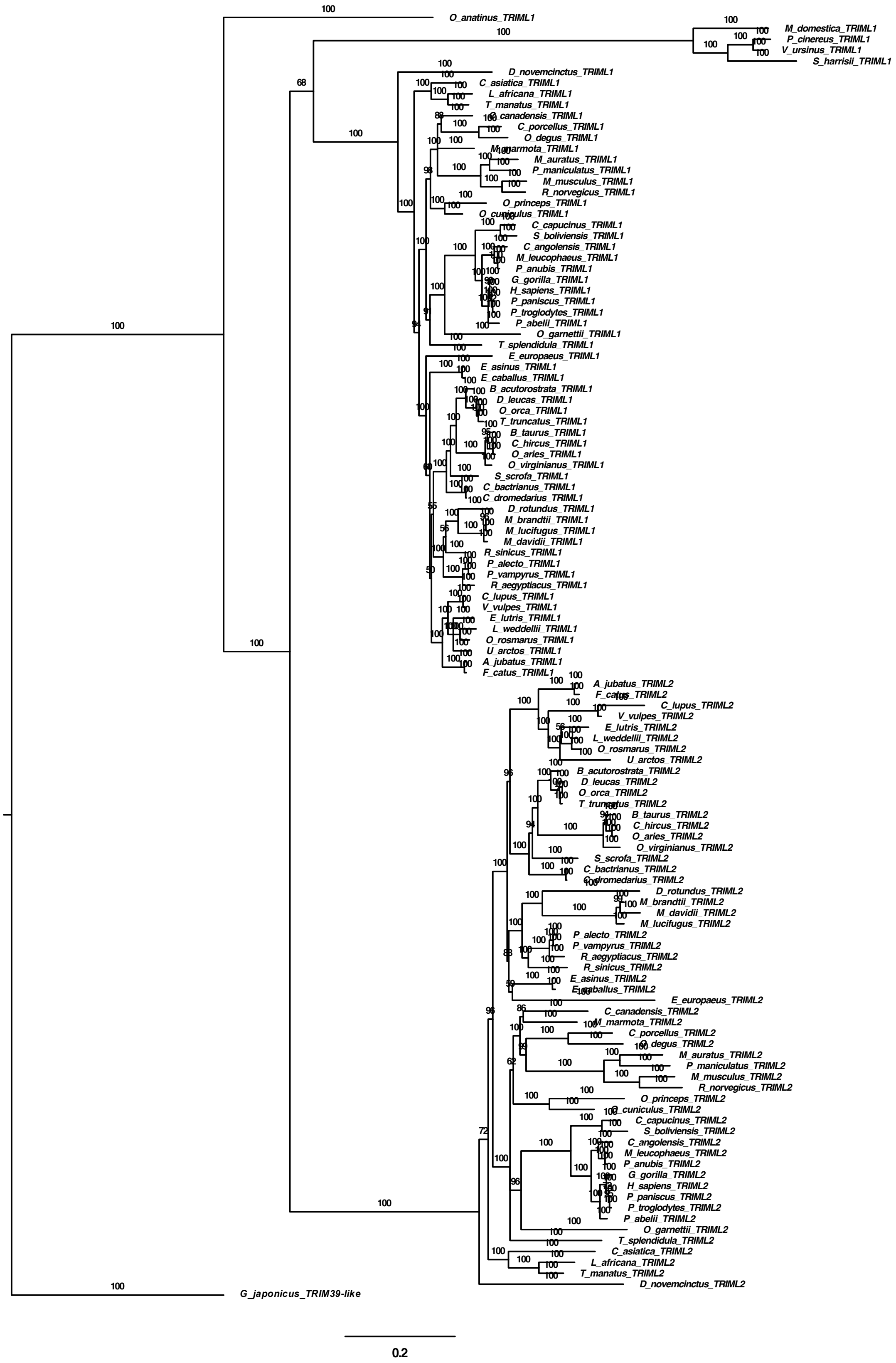
O_anatinus_TRIML1
M_domestica_TRIML1
P_cinereus_TRIML1
V_ursinus_TRIML1
S_harrisii_TRIML1
D_novemcinctus_TRIML1
C_asiatica_TRIML1
L_africana_TRIML1
T_manatus_TRIML1
C_canadensis_TRIML1
C_porcellus_TRIML1
O_degus_TRIML1
M_marmota_TRIML1
M_auratus_TRIML1
P_maniculatus_TRIML1
M_musculus_TRIML1
R_norvegicus_TRIML1
O_princeps_TRIML1
O_cuniculus_TRIML1
C_capucinus_TRIML1
S_boliviensis_TRIML1
C_angolensis_TRIML1
M_leucophaeus_TRIML1
P_anubis_TRIML1
G_gorilla_TRIML1
H_sapiens_TRIML1
P_paniscus_TRIML1
P_troglodytes_TRIML1
P_abelii_TRIML1
O_garnettii_TRIML1
T_splendidula_TRIML1
E_europaeus_TRIML1
E_asinus_TRIML1
E_caballus_TRIML1
B_acutorostrata_TRIML1
D_leucas_TRIML1
O_orca_TRIML1
T_truncatus_TRIML1
B_taurus_TRIML1
C_hircus_TRIML1
O_aries_TRIML1
O_virginianus_TRIML1
S_scrofa_TRIML1
C_bactrianus_TRIML1
C_dromedarius_TRIML1
D_rotundus_TRIML1
M_brandtii_TRIML1
M_lucifugus_TRIML1
M_davidii_TRIML1
R_sinicus_TRIML1
P_alecto_TRIML1
P_vampyrus_TRIML1
R_aegyptiacus_TRIML1
C_lupus_TRIML1
V_vulpes_TRIML1
E_lutris_TRIML1
L_weddellii_TRIML1
O_rosmarus_TRIML1
U_arctos_TRIML1
A_jubatus_TRIML1
F_catus_TRIML1
A_jubatus_TRIML2
F_catus_TRIML2
C_lupus_TRIML2
V_vulpes_TRIML2
E_lutris_TRIML2
L_weddellii_TRIML2
O_rosmarus_TRIML2
U_arctos_TRIML2
B_acutorostrata_TRIML2
D_leucas_TRIML2
O_orca_TRIML2
T_truncatus_TRIML2
B_taurus_TRIML2
C_hircus_TRIML2
O_aries_TRIML2
O_virginianus_TRIML2
S_scrofa_TRIML2
C_bactrianus_TRIML2
C_dromedarius_TRIML2
D_rotundus_TRIML2
M_brandtii_TRIML2
M_davidii_TRIML2
M_lucifugus_TRIML2
P_alecto_TRIML2
P_vampyrus_TRIML2
R_aegyptiacus_TRIML2
R_sinicus_TRIML2
E_asinus_TRIML2
E_caballus_TRIML2
E_europaeus_TRIML2
C_canadensis_TRIML2
M_marmota_TRIML2
C_porcellus_TRIML2
O_degus_TRIML2
M_auratus_TRIML2
P_maniculatus_TRIML2
M_musculus_TRIML2
R_norvegicus_TRIML2
O_princeps_TRIML2
O_cuniculus_TRIML2
C_capucinus_TRIML2
S_boliviensis_TRIML2
C_angolensis_TRIML2
M_leucophaeus_TRIML2
P_anubis_TRIML2
G_gorilla_TRIML2
H_sapiens_TRIML2
P_paniscus_TRIML2
P_troglodytes_TRIML2
P_abelii_TRIML2
O_garnettii_TRIML2
T_splendidula_TRIML2
C_asiatica_TRIML2
L_africana_TRIML2
T_manatus_TRIML2
D_novemcinctus_TRIML2
G_japonicus_TRIM39-like
0.2

C.

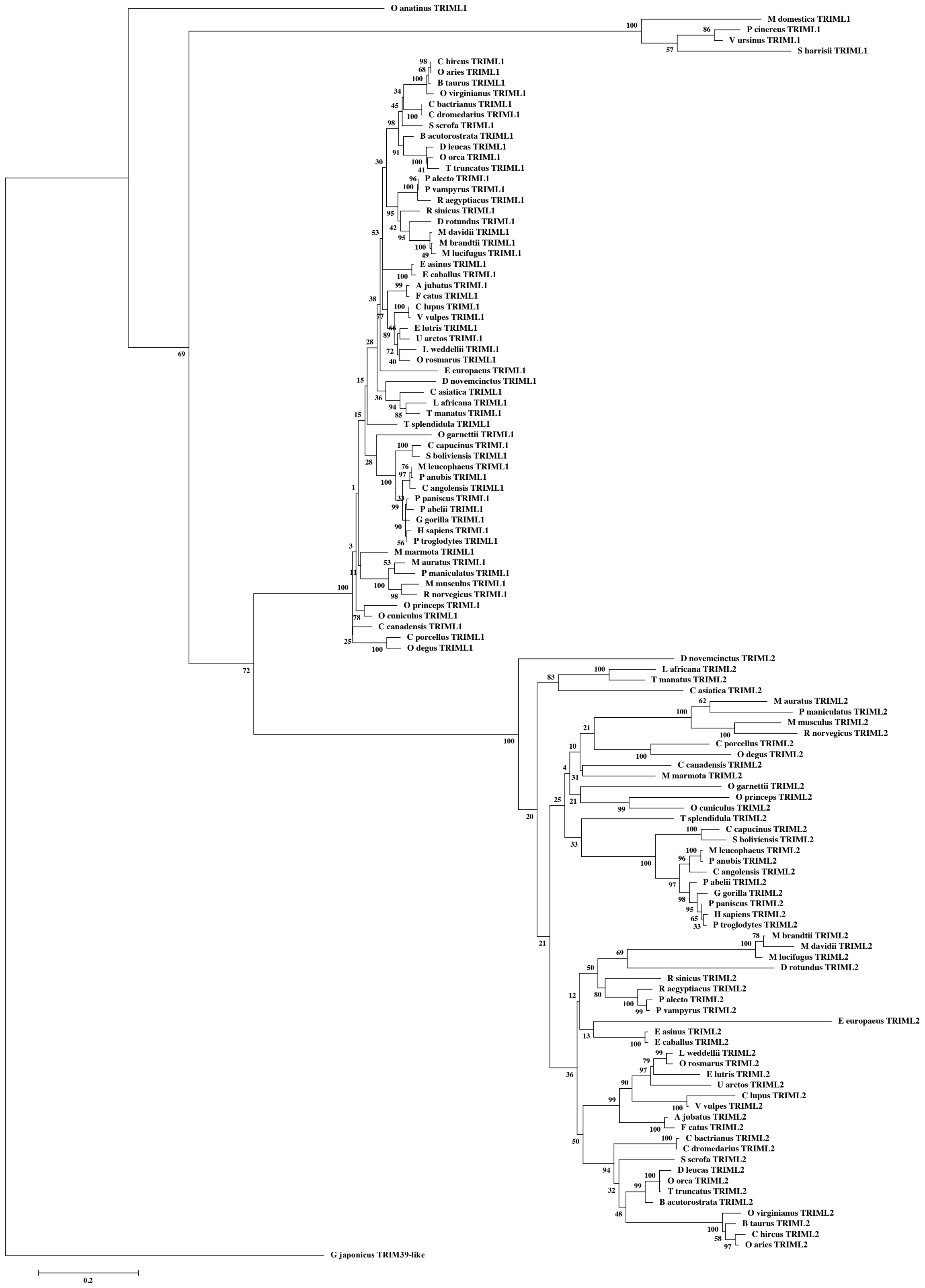
O anatinus TRIML1
M domestica TRIML1
P cinereus TRIML1
V ursinus TRIML1
S harrisii TRIML1
C hircus TRIML1
O aries TRIML1
B taurus TRIML1
O virginianus TRIML1
C bactrianus TRIML1
C dromedarius TRIML1
S scrofa TRIML1
B acutorostrata TRIML1
D leucas TRIML1
O orca TRIML1
T truncatus TRIML1
P alecto TRIML1
P vampyrus TRIML1
R aegyptiacus TRIML1
R sinicus TRIML1
D rotundus TRIML1
M davidii TRIML1
M brandtii TRIML1
M lucifugus TRIML1
E asinus TRIML1
E caballus TRIML1
A jubatus TRIML1
F catus TRIML1
C lupus TRIML1
V vulpes TRIML1
E lutris TRIML1
U arctos TRIML1
L weddellii TRIML1
O rosmarus TRIML1
E europaeus TRIML1
D novemcinctus TRIML1
C asiatica TRIML1
L africana TRIML1
T manatus TRIML1
T splendidula TRIML1
O garnettii TRIML1
C capucinus TRIML1
S boliviensis TRIML1
M leucophaeus TRIML1
P anubis TRIML1
C angolensis TRIML1
P paniscus TRIML1
P abelii TRIML1
G gorilla TRIML1
H sapiens TRIML1
P troglodytes TRIML1
M marmota TRIML1
M auratus TRIML1
P maniculatus TRIML1
M musculus TRIML1
R norvegicus TRIML1
O princeps TRIML1
O cuniculus TRIML1
C canadensis TRIML1
C porcellus TRIML1
O degus TRIML1
D novemcinctus TRIML2
L africana TRIML2
T manatus TRIML2
C asiatica TRIML2
M auratus TRIML2
P maniculatus TRIML2
M musculus TRIML2
R norvegicus TRIML2
C porcellus TRIML2
O degus TRIML2
C canadensis TRIML2
M marmota TRIML2
O garnettii TRIML2
O princeps TRIML2
O cuniculus TRIML2
T splendidula TRIML2
C capucinus TRIML2
S boliviensis TRIML2
M leucophaeus TRIML2
P anubis TRIML2
C angolensis TRIML2
P abelii TRIML2
G gorilla TRIML2
P paniscus TRIML2
H sapiens TRIML2
P troglodytes TRIML2
M brandtii TRIML2
M davidii TRIML2
M lucifugus TRIML2
D rotundus TRIML2
R sinicus TRIML2
R aegyptiacus TRIML2
P alecto TRIML2
P vampyrus TRIML2
E europaeus TRIML2
E asinus TRIML2
E caballus TRIML2
L weddellii TRIML2
O rosmarus TRIML2
E lutris TRIML2
U arctos TRIML2
C lupus TRIML2
V vulpes TRIML2
A jubatus TRIML2
F catus TRIML2
C bactrianus TRIML2
C dromedarius TRIML2
S scrofa TRIML2
D leucas TRIML2
O orca TRIML2
T truncatus TRIML2
B acutorostrata TRIML2
O virginianus TRIML2
B taurus TRIML2
C hircus TRIML2
O aries TRIML2
G japonicus TRIM39-like
0.2

D.

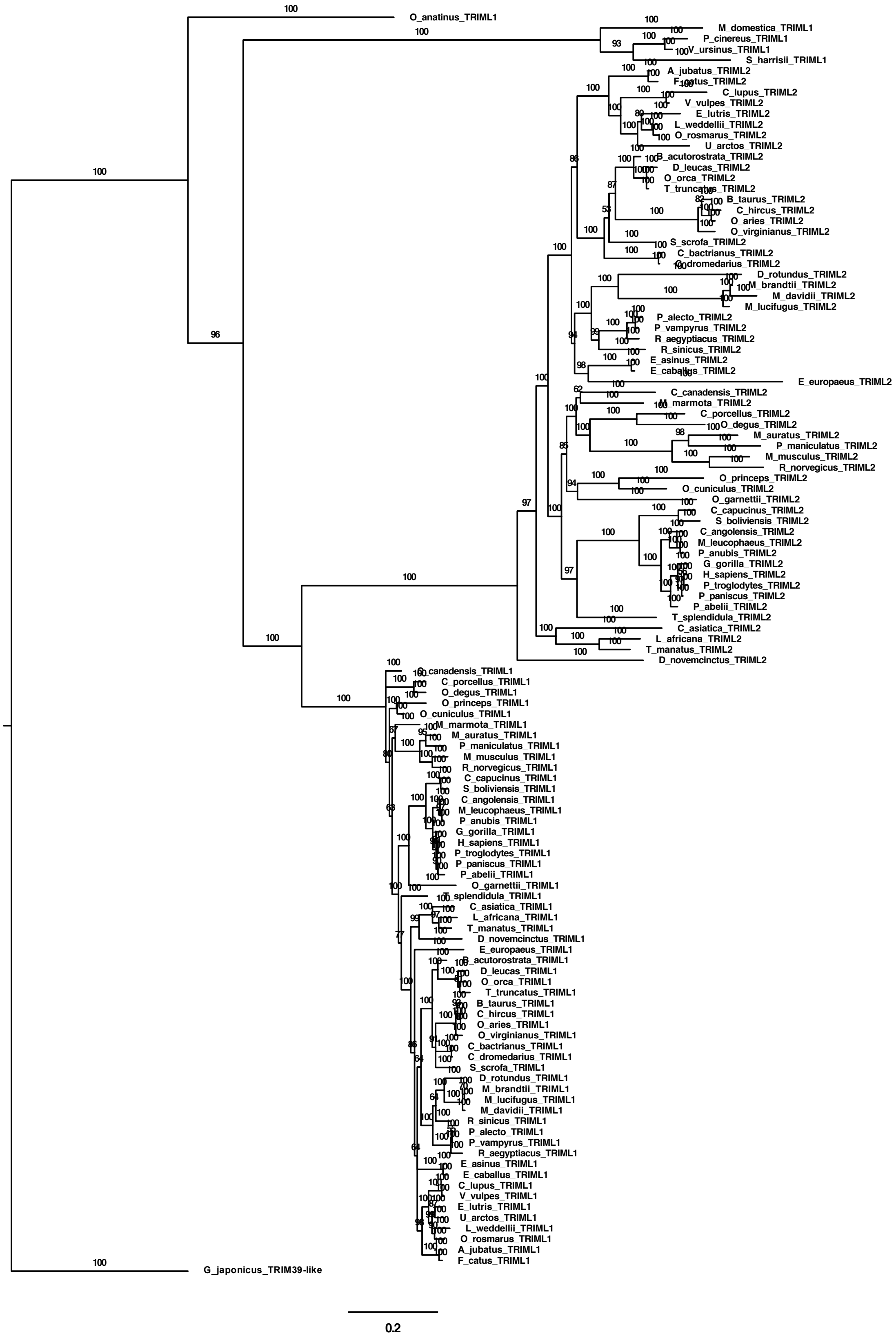
O_anatinus_TRIML1
M_domestica_TRIML1
P_cinereus_TRIML1
V_ursinus_TRIML1
S_harrisii_TRIML1
A_jubatus_TRIML2
F_catus_TRIML2
C_lupus_TRIML2
V_vulpes_TRIML2
E_lutris_TRIML2
L_weddellii_TRIML2
O_rosmarus_TRIML2
U_arctos_TRIML2
B_acutorostrata_TRIML2
D_leucas_TRIML2
O_orca_TRIML2
T_truncatus_TRIML2
B_taurus_TRIML2
C_hircus_TRIML2
O_aries_TRIML2
O_virginianus_TRIML2
S_scrofa_TRIML2
C_bactrianus_TRIML2
C_dromedarius_TRIML2
D_rotundus_TRIML2
M_brandtii_TRIML2
M_davidii_TRIML2
M_lucifugus_TRIML2
P_alecto_TRIML2
P_vampyrus_TRIML2
R_aegyptiacus_TRIML2
R_sinicus_TRIML2
E_asinus_TRIML2
E_caballus_TRIML2
E_europaeus_TRIML2
C_canadensis_TRIML2
M_marmota_TRIML2
C_porcellus_TRIML2
O_degus_TRIML2
M_auratus_TRIML2
P_maniculatus_TRIML2
M_musculus_TRIML2
R_norvegicus_TRIML2
O_princeps_TRIML2
O_cuniculus_TRIML2
O_garnettii_TRIML2
C_capucinus_TRIML2
S_boliviensis_TRIML2
C_angolensis_TRIML2
M_leucophaeus_TRIML2
P_anubis_TRIML2
G_gorilla_TRIML2
H_sapiens_TRIML2
P_troglodytes_TRIML2
P_paniscus_TRIML2
P_abelii_TRIML2
T_splendidula_TRIML2
C_asiatica_TRIML2
L_africana_TRIML2
T_manatus_TRIML2
D_novemcinctus_TRIML2
C_canadensis_TRIML1
C_porcellus_TRIML1
O_degus_TRIML1
O_princeps_TRIML1
O_cuniculus_TRIML1
M_marmota_TRIML1
M_auratus_TRIML1
P_maniculatus_TRIML1
M_musculus_TRIML1
R_norvegicus_TRIML1
C_capucinus_TRIML1
S_boliviensis_TRIML1
C_angolensis_TRIML1
M_leucophaeus_TRIML1
P_anubis_TRIML1
G_gorilla_TRIML1
H_sapiens_TRIML1
P_troglodytes_TRIML1
P_paniscus_TRIML1
P_abelii_TRIML1
O_garnettii_TRIML1
T_splendidula_TRIML1
C_asiatica_TRIML1
L_africana_TRIML1
T_manatus_TRIML1
D_novemcinctus_TRIML1
E_europaeus_TRIML1
B_acutorostrata_TRIML1
D_leucas_TRIML1
O_orca_TRIML1
T_truncatus_TRIML1
B_taurus_TRIML1
C_hircus_TRIML1
O_aries_TRIML1
O_virginianus_TRIML1
C_bactrianus_TRIML1
C_dromedarius_TRIML1
S_scrofa_TRIML1
D_rotundus_TRIML1
M_brandtii_TRIML1
M_lucifugus_TRIML1
M_davidii_TRIML1
R_sinicus_TRIML1
P_alecto_TRIML1
P_vampyrus_TRIML1
R_aegyptiacus_TRIML1
E_asinus_TRIML1
E_caballus_TRIML1
C_lupus_TRIML1
V_vulpes_TRIML1
E_lutris_TRIML1
U_arctos_TRIML1
L_weddellii_TRIML1
O_rosmarus_TRIML1
A_jubatus_TRIML1
F_catus_TRIML1
G_japonicus_TRIM39-like
0.2

E.

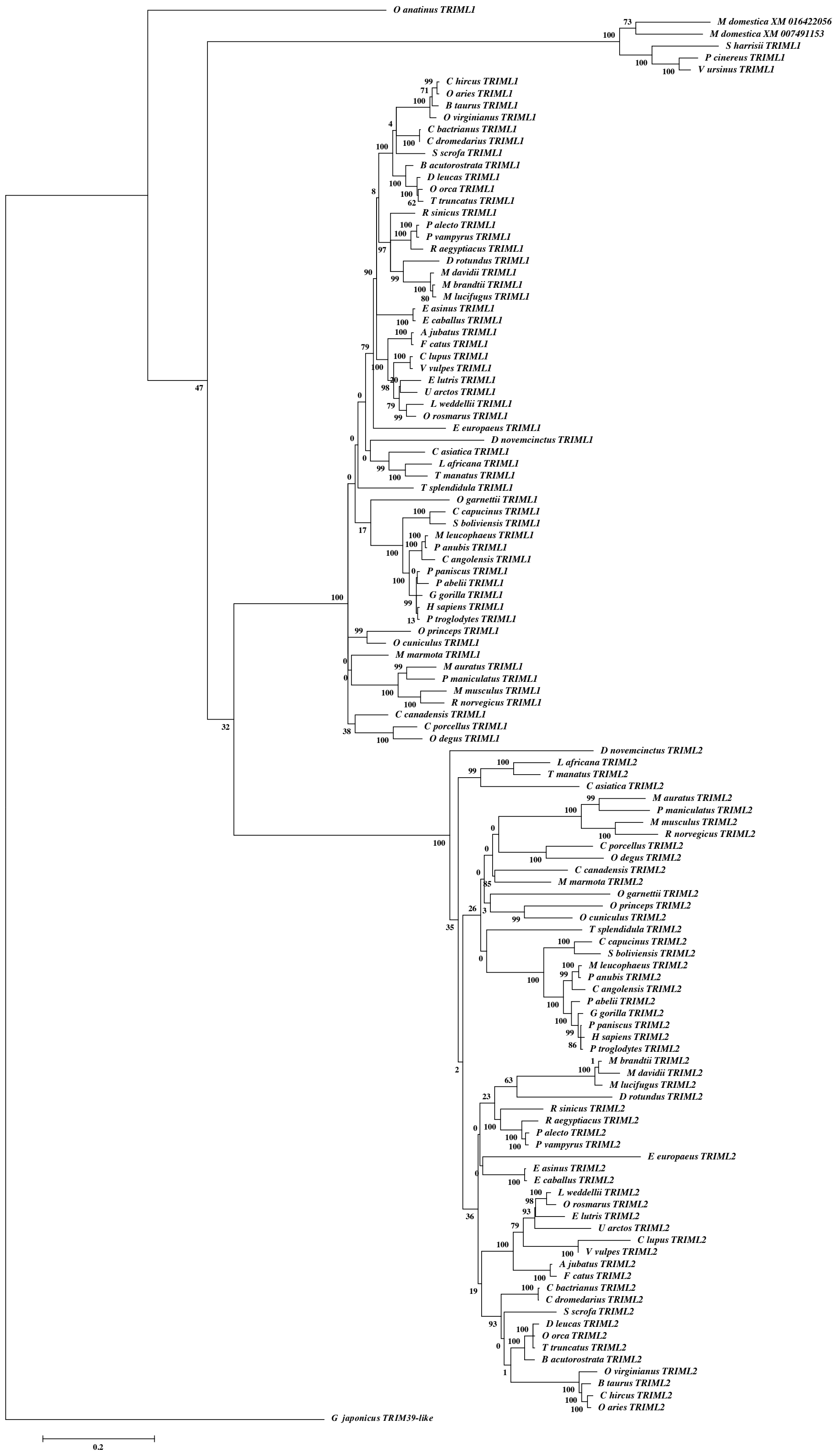

O anatinus TRIML1
M domestica XM 016422056
M domestica XM 007491153
S harrisii TRIML1
P cinereus TRIML1
V ursinus TRIML1
C hircus TRIML1
O aries TRIML1
B taurus TRIML1
O virginianus TRIML1
C bactrianus TRIML1
C dromedarius TRIML1
S scrofa TRIML1
B acutorostrata TRIML1
D leucas TRIML1
O orca TRIML1
T truncatus TRIML1
R sinicus TRIML1
P alecto TRIML1
P vampyrus TRIML1
R aegyptiacus TRIML1
D rotundus TRIML1
M davidii TRIML1
M brandtii TRIML1
M lucifugus TRIML1
E asinus TRIML1
E caballus TRIML1
A jubatus TRIML1
F catus TRIML1
C lupus TRIML1
V vulpes TRIML1
E lutris TRIML1
U arctos TRIML1
L weddellii TRIML1
O rosmarus TRIML1
E europaeus TRIML1
D novemcinctus TRIML1
C asiatica TRIML1
L africana TRIML1
T manatus TRIML1
T splendidula TRIML1
O garnettii TRIML1
C capucinus TRIML1
S boliviensis TRIML1
M leucophaeus TRIML1
P anubis TRIML1
C angolensis TRIML1
P paniscus TRIML1
P abelii TRIML1
G gorilla TRIML1
H sapiens TRIML1
P troglodytes TRIML1
O princeps TRIML1
O cuniculus TRIML1
M marmota TRIML1
M auratus TRIML1
P maniculatus TRIML1
M musculus TRIML1
R norvegicus TRIML1
C canadensis TRIML1
C porcellus TRIML1
O degus TRIML1
D novemcinctus TRIML2
L africana TRIML2
T manatus TRIML2
C asiatica TRIML2
M auratus TRIML2
P maniculatus TRIML2
M musculus TRIML2
R norvegicus TRIML2
C porcellus TRIML2
O degus TRIML2
C canadensis TRIML2
M marmota TRIML2
O garnettii TRIML2
O princeps TRIML2
O cuniculus TRIML2
T splendidula TRIML2
C capucinus TRIML2
S boliviensis TRIML2
M leucophaeus TRIML2
P anubis TRIML2
C angolensis TRIML2
P abelii TRIML2
G gorilla TRIML2
P paniscus TRIML2
H sapiens TRIML2
P troglodytes TRIML2
M brandtii TRIML2
M davidii TRIML2
M lucifugus TRIML2
D rotundus TRIML2
R sinicus TRIML2
R aegyptiacus TRIML2
P alecto TRIML2
P vampyrus TRIML2
E europaeus TRIML2
E asinus TRIML2
E caballus TRIML2
L weddellii TRIML2
O rosmarus TRIML2
E lutris TRIML2
U arctos TRIML2
C lupus TRIML2
V vulpes TRIML2
A jubatus TRIML2
F catus TRIML2
C bactrianus TRIML2
C dromedarius TRIML2
S scrofa TRIML2
D leucas TRIML2
O orca TRIML2
T truncatus TRIML2
B acutorostrata TRIML2
O virginianus TRIML2
B taurus TRIML2
C hircus TRIML2
O aries TRIML2
G japonicus TRIM39-like
0.2

F.

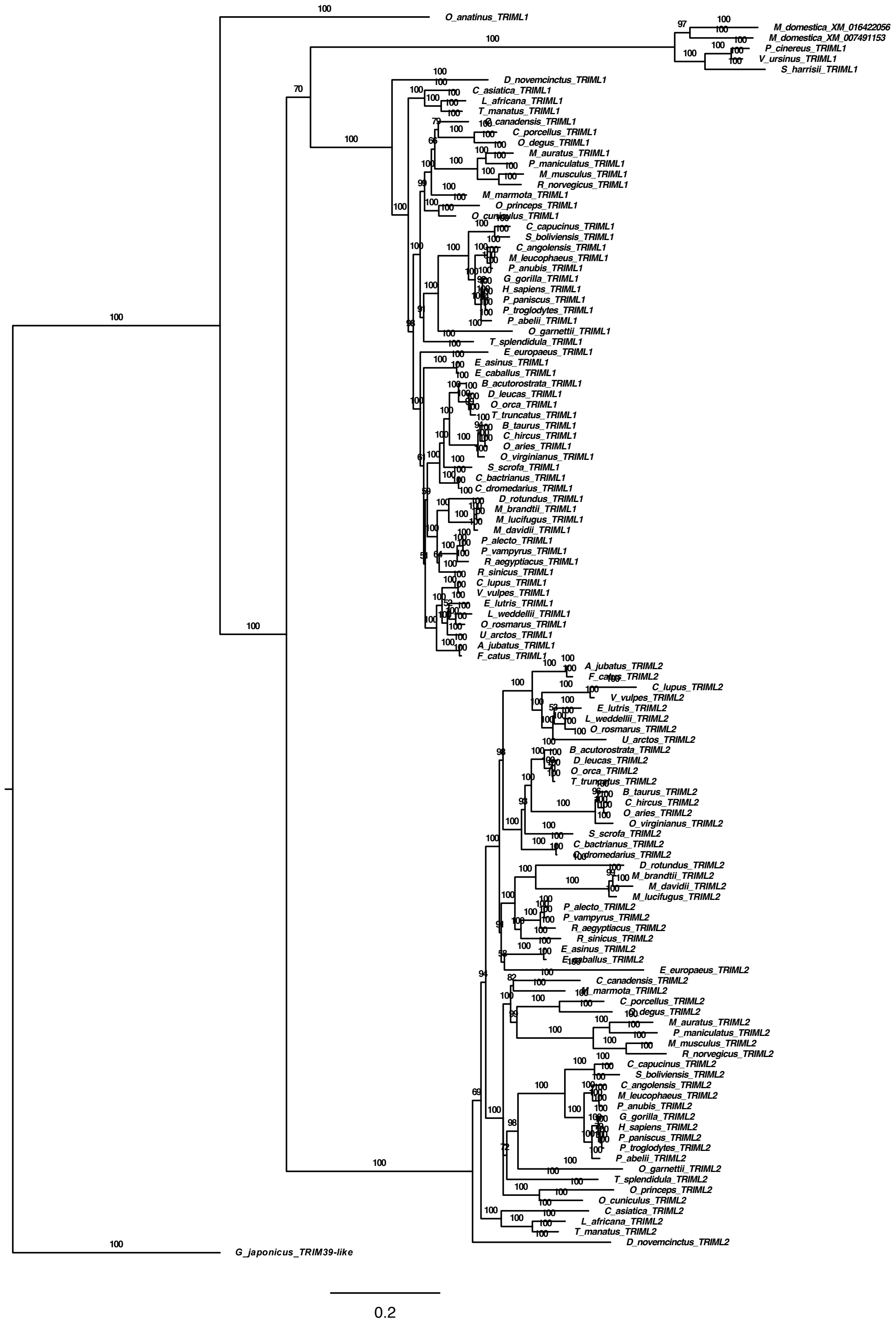
O_anatinus_TRIML1
M_domestica_XM_016422056
M_domestica_XM_007491153
P_cinereus_TRIML1
V_ursinus_TRIML1
S_harrisii_TRIML1
D_novemcinctus_TRIML1
C_asiatica_TRIML1
L_africana_TRIML1
T_manatus_TRIML1
C_canadensis_TRIML1
C_porcellus_TRIML1
O_degus_TRIML1
M_auratus_TRIML1
P_maniculatus_TRIML1
M_musculus_TRIML1
R_norvegicus_TRIML1
M_marmota_TRIML1
O_princeps_TRIML1
O_cuniculus_TRIML1
C_capucinus_TRIML1
S_boliviensis_TRIML1
C_angolensis_TRIML1
M_leucophaeus_TRIML1
P_anubis_TRIML1
G_gorilla_TRIML1
H_sapiens_TRIML1
P_paniscus_TRIML1
P_troglodytes_TRIML1
P_abelii_TRIML1
O_garnettii_TRIML1
T_splendidula_TRIML1
E_europaeus_TRIML1
E_asinus_TRIML1
E_caballus_TRIML1
B_acutorostrata_TRIML1
D_leucas_TRIML1
O_orca_TRIML1
T_truncatus_TRIML1
B_taurus_TRIML1
C_hircus_TRIML1
O_aries_TRIML1
O_virginianus_TRIML1
S_scrofa_TRIML1
C_bactrianus_TRIML1
C_dromedarius_TRIML1
D_rotundus_TRIML1
M_brandtii_TRIML1
M_lucifugus_TRIML1
M_davidii_TRIML1
P_alecto_TRIML1
P_vampyrus_TRIML1
R_aegyptiacus_TRIML1
R_sinicus_TRIML1
C_lupus_TRIML1
V_vulpes_TRIML1
E_lutris_TRIML1
L_weddellii_TRIML1
O_rosmarus_TRIML1
U_arctos_TRIML1
A_jubatus_TRIML1
F_catus_TRIML1
A_jubatus_TRIML2
F_catus_TRIML2
C_lupus_TRIML2
V_vulpes_TRIML2
E_lutris_TRIML2
L_weddellii_TRIML2
O_rosmarus_TRIML2
U_arctos_TRIML2
B_acutorostrata_TRIML2
D_leucas_TRIML2
O_orca_TRIML2
T_truncatus_TRIML2
B_taurus_TRIML2
C_hircus_TRIML2
O_aries_TRIML2
O_virginianus_TRIML2
S_scrofa_TRIML2
C_bactrianus_TRIML2
C_dromedarius_TRIML2
D_rotundus_TRIML2
M_brandtii_TRIML2
M_davidii_TRIML2
M_lucifugus_TRIML2
P_alecto_TRIML2
P_vampyrus_TRIML2
R_aegyptiacus_TRIML2
R_sinicus_TRIML2
E_asinus_TRIML2
E_caballus_TRIML2
E_europaeus_TRIML2
C_canadensis_TRIML2
M_marmota_TRIML2
C_porcellus_TRIML2
O_degus_TRIML2
M_auratus_TRIML2
P_maniculatus_TRIML2
M_musculus_TRIML2
R_norvegicus_TRIML2
C_capucinus_TRIML2
S_boliviensis_TRIML2
C_angolensis_TRIML2
M_leucophaeus_TRIML2
P_anubis_TRIML2
G_gorilla_TRIML2
H_sapiens_TRIML2
P_paniscus_TRIML2
P_troglodytes_TRIML2
P_abelii_TRIML2
O_garnettii_TRIML2
T_splendidula_TRIML2
O_princeps_TRIML2
O_cuniculus_TRIML2
C_asiatica_TRIML2
L_africana_TRIML2
T_manatus_TRIML2
D_novemcinctus_TRIML2
G_japonicus_TRIM39-like
0.2

G.

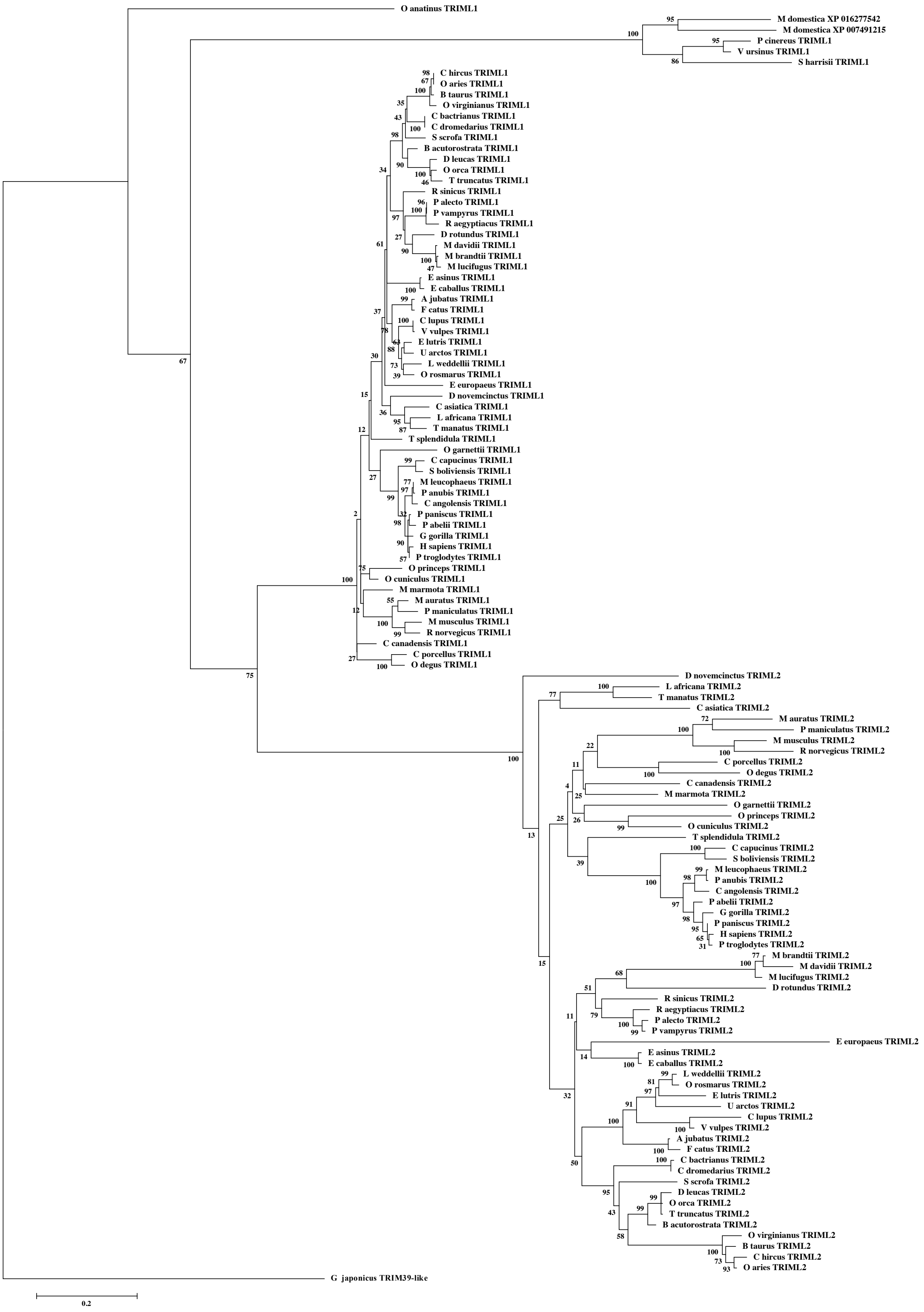
O anatinus TRIML1
M domestica XP 016277542
M domestica XP 007491215
P cinereus TRIML1
V ursinus TRIML1
S harrisii TRIML1
C hircus TRIML1
O aries TRIML1
B taurus TRIML1
O virginianus TRIML1
C bactrianus TRIML1
C dromedarius TRIML1
S scrofa TRIML1
B acutorostrata TRIML1
D leucas TRIML1
O orca TRIML1
T truncatus TRIML1
R sinicus TRIML1
P alecto TRIML1
P vampyrus TRIML1
R aegyptiacus TRIML1
D rotundus TRIML1
M davidii TRIML1
M brandtii TRIML1
M lucifugus TRIML1
E asinus TRIML1
E caballus TRIML1
A jubatus TRIML1
F catus TRIML1
C lupus TRIML1
V vulpes TRIML1
E lutris TRIML1
U arctos TRIML1
L weddellii TRIML1
O rosmarus TRIML1
E europaeus TRIML1
D novemcinctus TRIML1
C asiatica TRIML1
L africana TRIML1
T manatus TRIML1
T splendidula TRIML1
O garnettii TRIML1
C capucinus TRIML1
S boliviensis TRIML1
M leucophaeus TRIML1
P anubis TRIML1
C angolensis TRIML1
P paniscus TRIML1
P abelii TRIML1
G gorilla TRIML1
H sapiens TRIML1
P troglodytes TRIML1
O princeps TRIML1
O cuniculus TRIML1
M marmota TRIML1
M auratus TRIML1
P maniculatus TRIML1
M musculus TRIML1
R norvegicus TRIML1
C canadensis TRIML1
C porcellus TRIML1
O degus TRIML1
D novemcinctus TRIML2
L africana TRIML2
T manatus TRIML2
C asiatica TRIML2
M auratus TRIML2
P maniculatus TRIML2
M musculus TRIML2
R norvegicus TRIML2
C porcellus TRIML2
O degus TRIML2
C canadensis TRIML2
M marmota TRIML2
O garnettii TRIML2
O princeps TRIML2
O cuniculus TRIML2
T splendidula TRIML2
C capucinus TRIML2
S boliviensis TRIML2
M leucophaeus TRIML2
P anubis TRIML2
C angolensis TRIML2
P abelii TRIML2
G gorilla TRIML2
P paniscus TRIML2
H sapiens TRIML2
P troglodytes TRIML2
M brandtii TRIML2
M davidii TRIML2
M lucifugus TRIML2
D rotundus TRIML2
R sinicus TRIML2
R aegyptiacus TRIML2
P alecto TRIML2
P vampyrus TRIML2
E europaeus TRIML2
E asinus TRIML2
E caballus TRIML2
L weddellii TRIML2
O rosmarus TRIML2
E lutris TRIML2
U arctos TRIML2
C lupus TRIML2
V vulpes TRIML2
A jubatus TRIML2
F catus TRIML2
C bactrianus TRIML2
C dromedarius TRIML2
S scrofa TRIML2
D leucas TRIML2
O orca TRIML2
T truncatus TRIML2
B acutorostrata TRIML2
O virginianus TRIML2
B taurus TRIML2
C hircus TRIML2
O aries TRIML2
G japonicus TRIM39-like
0.2

H.

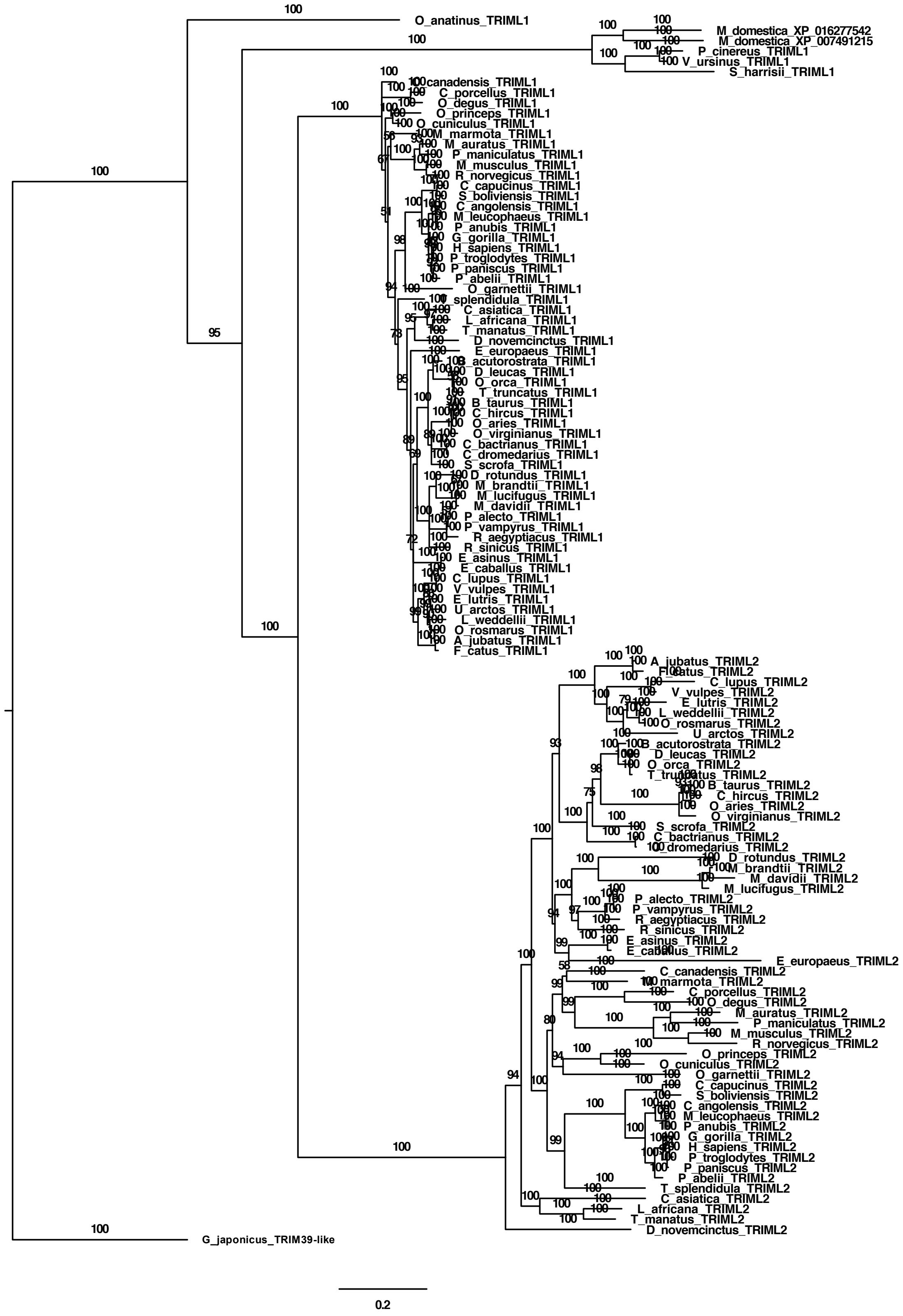
O_anatinus_TRIML1
M_domestica_XP_016277542
M_domestica_XP_007491215
P_cinereus_TRIML1
V_ursinus_TRIML1
S_harrisii_TRIML1
G_japonicus_TRIM39-like
0.2

Fig S3. Phylogenetic reconstruction of *TRIML1* and *TRIML2*. (A-D) phylogenetic trees generated with one opossum *TRIML1* (*XM_007491153*/ XP_ 007491215). (E-F) phylogenetic trees generated with both potential opossum *TRIML1*s. (A, E) Phylogenetic trees generated using maximum likelihood (ML) approach, based on mRNA sequences. (B, F) Phylogenetic trees generated using Bayesian analysis, based on mRNA sequences. (C, G) Phylogenetic trees generated using ML, based on protein sequences. (D, H) Phylogenetic trees generated using Bayesian analysis, based on protein sequences. Bootstrap percentages / posterior probabilities are labeled.

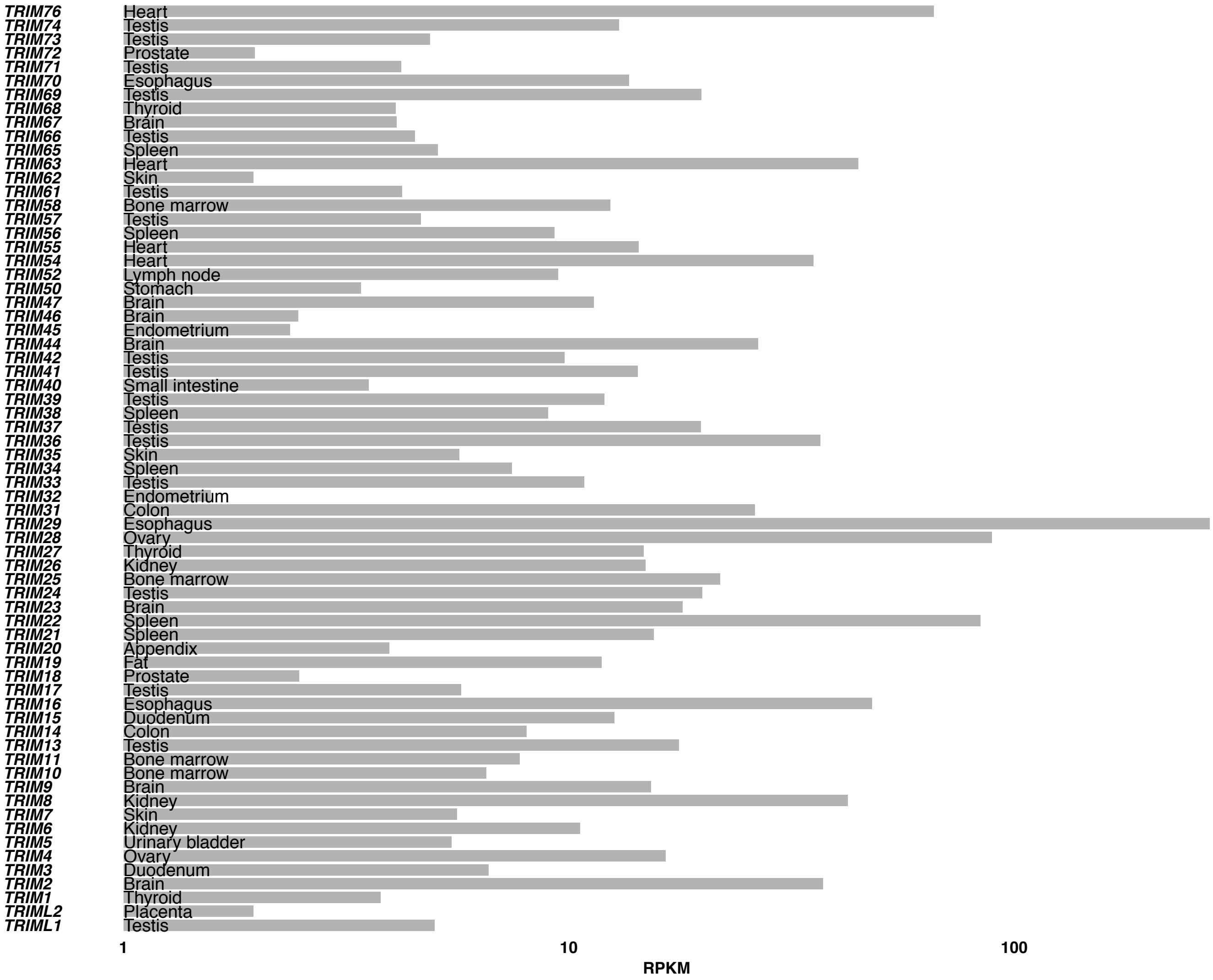

Fig S4. Tissue with highest expression for each *TRIM* gene (data from the Human Protein Atlas).

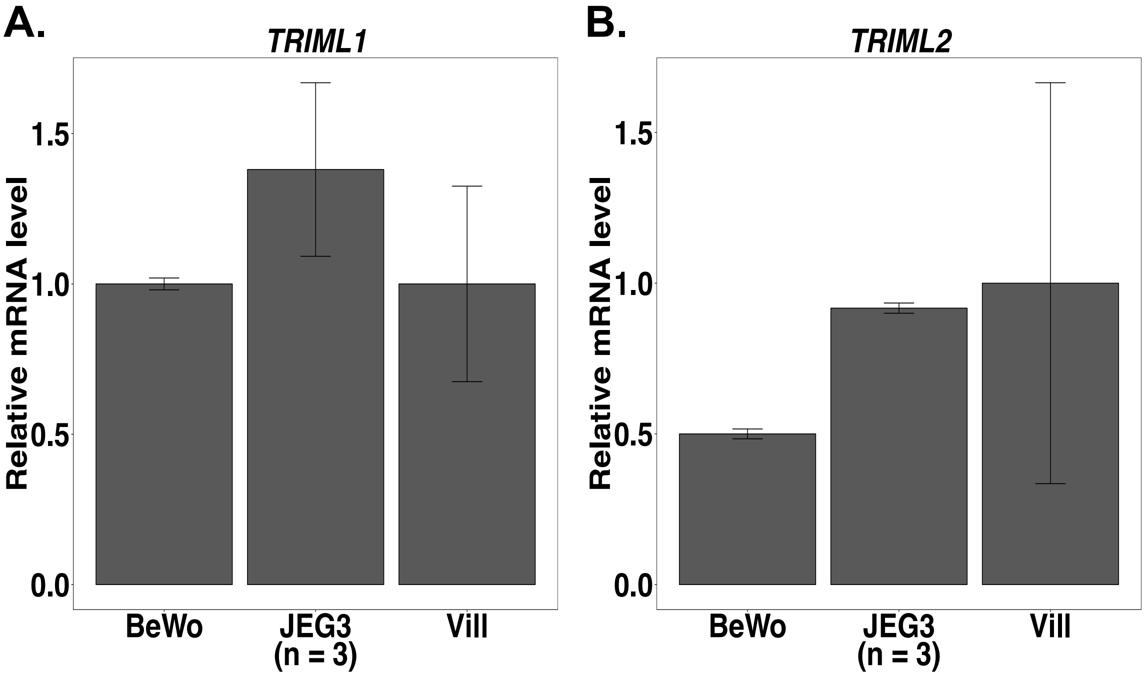

Fig S5. *TRIML1* and *TRIML2* mRNA expression in BeWo cells and JEG3 cells. Expression levels normalized to term human placenta villi. Vill, placental villi. Values are mean ± SEM.
